# Probing Single-Cell Fermentation Fluxes and Exchange Networks via pH-Sensing Hybrid Nanofibers

**DOI:** 10.1021/acsnano.2c06114

**Published:** 2022-12-27

**Authors:** Valentina Onesto, Stefania Forciniti, Francesco Alemanno, Krishnadev Narayanankutty, Anil Chandra, Saumya Prasad, Amalia Azzariti, Giuseppe Gigli, Adriano Barra, Andrea De Martino, Daniele De Martino, Loretta L. del Mercato

**Affiliations:** † Institute of Nanotechnology, National Research Council (CNR-NANOTEC), c/o Campus Ecotekne, via Monteroni, 73100Lecce, Italy; ‡ Dipartimento di Matematica e Fisica E. De Giorgi, University of Salento, 73100Lecce, Italy; § Istituto Nazionale di Fisica Nucleare (INFN), Sezione di Lecce, 73100Lecce, Italy; ⊥ Biofisika Institutua (UPV/EHU, CSIC) and Fundación Biofísica Bizkaia, LeioaE-48940, Spain; ∥ IRCCS Istituto Tumori Giovanni Paolo II, V.le O. Flacco, 65, 70124Bari, Italy; ¶ Politecnico di Torino, Corso Duca degli Abruzzi, 24, I-10129Torino, Italy; # Italian Institute for Genomic Medicine, IRCCS Candiolo, SP-142, I-10060Candiolo, Italy; ▼ Ikerbasque Foundation, Bilbao48013, Spain

**Keywords:** single-cell metabolism, Warburg effect, silica microparticles, nanofibers, pH sensing, fluorescence, inverse modeling

## Abstract

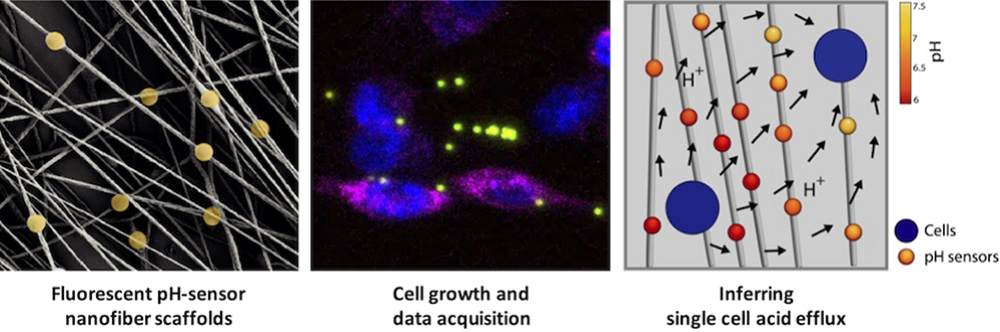

The homeostatic control of their environment is an essential task of living cells. It has been hypothesized that, when microenvironmental pH inhomogeneities are induced by high cellular metabolic activity, diffusing protons act as signaling molecules, driving the establishment of exchange networks sustained by the cell-to-cell shuttling of overflow products such as lactate. Despite their fundamental role, the extent and dynamics of such networks is largely unknown due to the lack of methods in single-cell flux analysis. In this study, we provide direct experimental characterization of such exchange networks. We devise a method to quantify single-cell fermentation fluxes over time by integrating high-resolution pH microenvironment sensing via ratiometric nanofibers with constraint-based inverse modeling. We apply our method to cell cultures with mixed populations of cancer cells and fibroblasts. We find that the proton trafficking underlying bulk acidification is strongly heterogeneous, with maximal single-cell fluxes exceeding typical values by up to 3 orders of magnitude. In addition, a crossover in time from a networked phase sustained by densely connected “hubs” (corresponding to cells with high activity) to a sparse phase dominated by isolated dipolar motifs (i.e., by pairwise cell-to-cell exchanges) is uncovered, which parallels the time course of bulk acidification. Our method addresses issues ranging from the homeostatic function of proton exchange to the metabolic coupling of cells with different energetic demands, allowing for real-time noninvasive single-cell metabolic flux analysis.

## Introduction

Fermentative processes are among the main modes of harnessing energy by cells. Despite their importance and the time elapsed since their discovery, they still continue to puzzle in regard to their basic function and mechanisms.^1^ Since the earliest observation of fermentation inhibition by oxygen^2^ and given their substantially lower efficiency with respect to oxidative pathways, fermentative pathways were initially seen as evolutionary relics with a subsidiary role, to be employed mainly in anoxygenic conditions and with problematic waste byproducts. Subsequent observations, however, established their ubiquitous usage, even in the presence of oxygen and especially for high energetic loads (e.g., during fast cellular growth), a phenomenon now known as “overflow metabolism”.^3^ Because of its universality, current research efforts have been devoted to understanding fermentation mechanism and function, e.g., in terms of volume constraints^4,5^ and/or temperature homeostasis.^6^ A fundamental aspect of fermentative activity lies in its ecological dimension, specifically in its capability to alter the cellular microenvironment and most notably the pH level.

**Figure 1:**
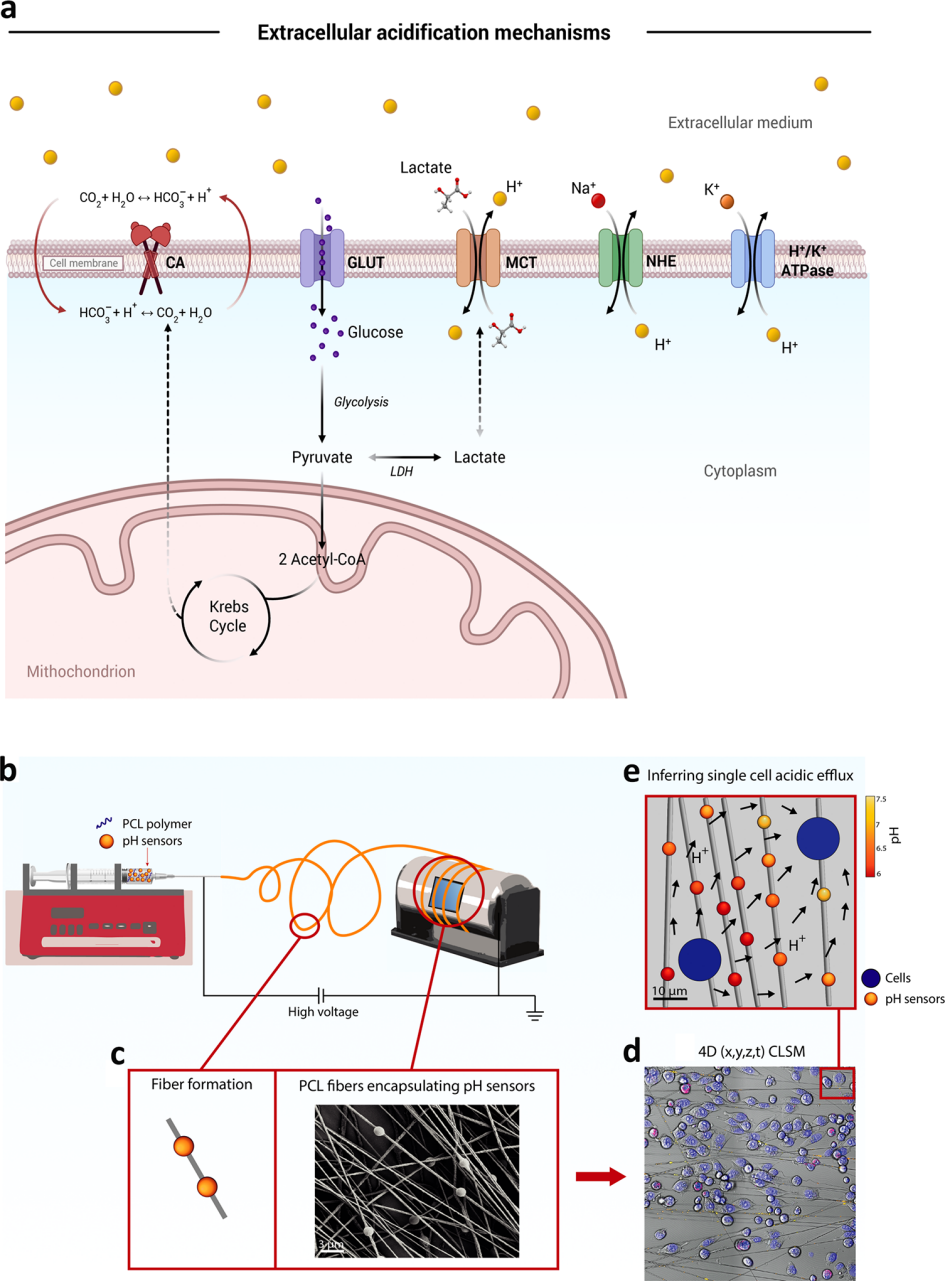
(a) Sketch of the main processes, enzymes and transporters involved in the proton exchanges with the extracellular medium. (b) Sketch showing the fabrication of electrospun polycaprolactone (PCL) fibers embedding ratiometric SiO_2_-based microparticle sensors. (c) Representative SEM micrograph showing the morphology of PCL nonwoven mat of fibers carrying embedded pH sensors. (d) Representative CLSM image showing cells cocultured on pH-sensing fibers and analyzed by CLSM time lapse imaging (*x*, *y*, *z*, *t*; *t* = 6 h) (nuclei are shown in blue, and cell membranes are shown in magenta for tumor cells). (e) Following spatial tracking of cells and probes, the whole pH gradient and the boundary single-cell fluxes are reconstructed through physically constrained statistical inference.

Cells dispose of a number of mechanisms to control their internal pH, which varies significantly across compartments (from ∼8 in the mitochondrial matrix down to about 5 in secretory granules^7^). Cytoplasmic pH is especially impacted by the membrane potential (which tends to let positive ions in and negative ions out) and by the metabolic activity of the cell. Most notably, energy production by glycolysis, which occurs at high rates e.g., in cancer, generates cytosol-acidifying lactate. To counter this tendency to reduce cytosolic pH, cells utilize an array of transporters that couple proton export to the export (cotransporters) or import (exchangers) of some other metabolite, and whose activity is kinetically regulated by sites acting effectively as pH sensors^7^ (Figure 1a). When present, lactate is actively cotransported out of the cell by monocarboxylate transporters (MCT) with a proton,^8^ which in turn acidifies the extracellular space to a pH between 6.2 and 6.9. As CO_2_ easily penetrates the plasma membrane and water leaves the cell through aquaporins, transmembrane carbonic anhydrases (CA) contribute to acid/base homeostasis by typically favoring proton/bicarbonate formation in the extracellular space.^9^ Na^+^/H^+^ exchangers (NHE) are normally activated by stress factors like hypoxia.^10^ Hydrogen/potassium (H–K) ATPase^11^ are instead the main drivers of gastric acidification and are also expressed in pancreatic cells. On top of this, extracellular pH can be regulated via secondary effects through a complex signaling network driven by protons and lactate. For instance, an increase in lactate concentration in the tumor extracellular space suppresses lactate excretion by other cells that rely on glycolysis for energy synthesis.^12^ This plethora of processes induces a reduction of the pH of the surrounding medium, which is in turn sensed by nearby cells. To maintain a stable extracellular proton level, then, these cells can respond by activating specific H^+^-sensitive channels and receptors.

The excretion of lactate by cells with large energetic demands and carbon consumptions, like tumors (the so-called Warburg effect^13^) or neurons,^14^ constitutes an especially interesting case of microenvironmental acidification. Indeed, while the intracellular (respectively extracellular) pH of normal cells lies roughly in the range 6.99–7.05 (respectively 7.35–7.45), that of tumor cells varies instead between 7.12–7.7 (respectively 6.2–6.9). In other words, in normal cells, intracellular pH is lower than extracellular pH; and vice versa, in tumor cells, intracellular pH is higher than extracellular pH. To maintain this inverse pH gradient, lactate-secreting cells may rely on metabolic partnerships with their non-lactate-secreting neighbors. It has indeed been hypothesized that lactate might become a fundamental vector of metabolic coupling within cellular populations in higher organisms:^15,16^ lactate-importing cells (acceptors) could rely on lactate-secreting cells (donors) for subsistence, while providing an essential bioremediation function by removing an acidifying metabolite from the microenvironment.^17,18^ This idea adds an “ecologic” dimension to pH-driven intercellular communication.^19^

While molecular mechanisms behind proton sensing, export and import are by now rather well characterized in a number of systems, due to their pathophysiological role for tumorigenesis and in the mammalian brain,^20,21^ much less is known about the actual intercellular proton-exchange network that is established. The major obstacle to overcome concerns the quantification of proton exchange fluxes for single cells within a population. Techniques to quantify cellular metabolic fluxes are in general well developed for the bulk of (mostly microbial) cell cultures.^22−24^ Single-cell metabolomics, on the other hand, is less developed,^25^ with the exceptions of the growth rate,^26^ glucose uptake^27^ and more in general nanoSIMS-based analyses,^28,29^ whose destructive character is however a serious shortcoming.

In this study, we gather information about the proton-exchange network by combining pH microenvironment sensing via recently devised pH-sensing ratiometric hybrid organic nanofibers^30−32^ with constraint-based statistical inference. A sketch of the complete setup is shown in Figure 1b. It initially involves the electrospinning onto glass slides (10 mm × 10 mm), positioned on a custom rotating collecting system, of a 10% (w/w) polycaprolactone (PCL) chloroform/DMSO solution mixed with spherical and monodispersed SiO_2_ microparticle-based pH sensors (Figure 1c,d). In the second step, pancreatic ductal adenocarcinoma cells (PDAC cell line, AsPC-1) and pancreatic stellate cancer-associated fibroblasts (CAFs) are seeded onto the hybrid nanofibers and the fluorescence response of the pH sensors during cell culture is recorded via time lapse confocal laser scanning microscopy (CLSM) (Figure 1d). The third step involves precise automated quantification of intercellular proton (H^+^) exchange through physically constrained statistical inference (Figure 1e).

In addition to the accuracy of reconstructed fluxes, our method has the advantage of maintaining the sample intact. One can therefore monitor real-time single-cell behavior potentially up to the whole population scale.

After illustrating our screening platform, we will focus on a concrete application, namely the reconstruction of the proton exchange network that underlies bulk acidification in mixed populations of CAFs and PDAC cells (Warburg effect). Besides their specific relevance for the study of tumors, our choice for this coculture is motivated by its convenience: mixed populations of cancer cells and fibroblasts are expected to be ideally suited to expose the intercellular proton exchange network. Contrary to pure mammalian cell cultures, which can exhibit a negative feedback between the (potentially toxic) byproducts of metabolism (lactate in this case) and cellular fitness at fast enough growth,^33^ mixed populations rely on the establishment of extensive exchange interactions between cells to remediate the microenvironment while promoting viability and growth in both types.^18^ These results therefore address the ecology of cancer metabolism and, more generally, of metabolic overflow, while our method allows for noninvasive single-cell metabolic flux analysis.

## Results and Discussion

### Fabrication of pH-Sensing Scaffolds

**Figure 2:**
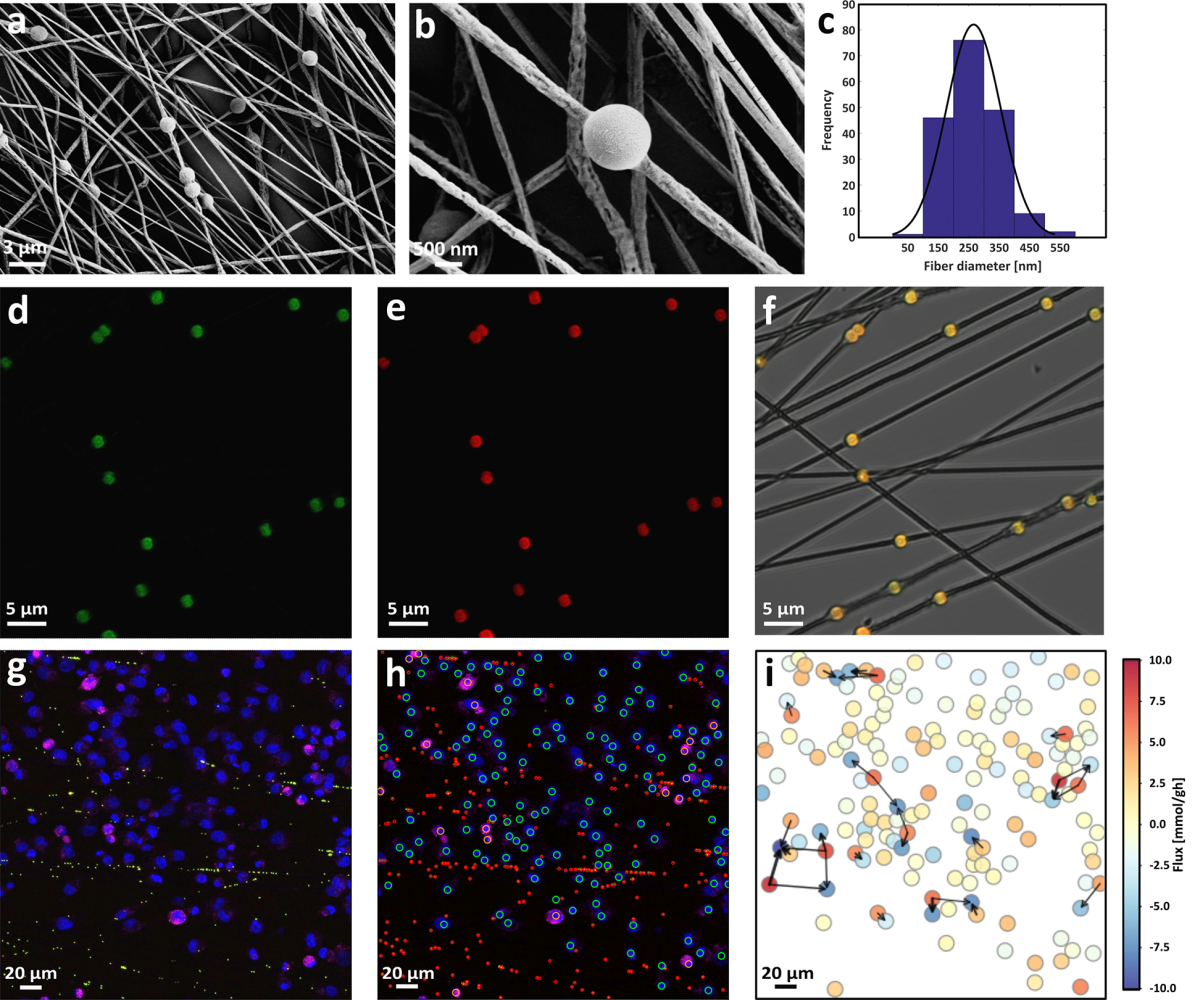
SEM micrographs showing the (a) pH sensors into the fiber’s lumen and (b) corrugated morphology of the surface of individual fibers (deposition time = 90 s). (c) Graph illustration of the diameter distribution of the hybrid nanofibers. The superimposed continuous line is the best-fitting Gaussian curve. (d–f) Representative CLSM micrographs showing PCL nanofibers embedding pH sensors (deposition time = 30 s). FITC (green channel), RBITC (red channel), and overlay with bright-field (BF, gray channel) are shown. (g) Representative images of CLSM time lapse image (maximum intensity projection) at the time point *t* = 3 h, showing pH-sensing particles (FITC, green; RBITC, red), AsPC-1 cells (Hoechst, blue; Deep Red, magenta), and CAF cells (Hoechst, blue). (h) Result of the segmentation of the experiment in (g) showing the detection of the single pH sensors (red circles), AsPC-1 cells (green circles), and CAF cells (yellow circles). (i) Reconstruction of the cell fluxes through physically constrained statistical inference, with a relative colormap.

We developed fluorescent pH-sensing nanofiber scaffolds composed of optical pH sensor microparticles, highly suitable for monitoring pH changes in the surrounding environment,^34−41^ and polycaprolactone (PCL) nanofibers, because of their processability, biocompatibility, biodegradability, and mechanical stability.^42−47^ The morphology of the hybrid pH-sensing fibers was studied in detail by means of scanning electron microscopy (SEM) and CLSM. The SEM images in Figure 2a,b show random nonwoven mat of PCL nanofibers bearing spherical pH sensors aligned along the fiber longitudinal axis. The fibers present a typical rough and porous surface structure (Figure 2b), likely due to the use of chloroform/DMSO binary solvent system.^48^ The PCL solution, as well as the concentration of the pH sensors and the electrospinning conditions were adjusted in order to obtain uniform and bead-free nanofibers with a controlled diameter of 272.43 ± 7.95 nm (Figure 2c) and a thickness of 2.91 ± 0.17 μm (Figure S1). Notably, these nanosized electrospun fiber scaffolds provide a large surface-to-volume ratio, which is known to enhance key cellular functions, including adhesion, proliferation, and differentiation.^49−51^ Moreover, their nanofibrous structure mimics the native extracellular matrix (ECM), which plays a pivotal role in cell polarity as well as in cell-to-cell/matrix interaction.^52−55^ Representative CLSM images of the pH-sensing nanofibers are shown in Figures 2d–f. Each pH sensor microparticle is clearly detectable thanks to the fluorescein 5(6)-isothiocyanate, FITC (Figure 2d), and Rhodamine B isothiocyanate, RBITC (Figure 2e), dye molecules covalently linked with APTES to the surface of silica (SiO_2_) microparticles.^56,57^ The FITC and RBITC fluorophores act as pH indicator and reference dyes, respectively, to enable ratiometric measurements of pH, which is a more robust and reliable way of fluorescence sensing because it compensates for fluctuation in fluorescence intensities due to the utilization of two distinct emission wavelengths.^58−61^ Thanks to the surrounding polymeric matrix, the pH sensors remain stably immobilized into the lumen of the nanofibers during imaging, making pH-sensing ratiometric hybrid nanofibers an ideal biomaterial scaffold for monitoring local microenvironment proton changes in a fast and noninvasive way, with high spatial control and resolution. The pH-sensing nanofibers were used to culture pancreatic cancer cells (AsPC-1) and pancreatic stellate cancer associated fibroblasts (CAFs) and to monitor extracellular pH changes via time lapse CLSM acquisitions for 6 h with time intervals of 10 min (Figure 2g). As shown in Figure S4, cell cocultures seeded on the pH-sensing nanofibers remained viable until the end of the time lapse experiment and for many days after (e.g., 8 days). CLSM acquisitions were analyzed through segmentation algorithms in order to identify cells and sensors within the images (Figure 2h), to quantify FITC/RBITC fluorescence intensity ratio and hence the pH read-outs. The acids efflux from each cell was obtained via statistical inference (Figure 2i) as described in detail in the following sections.

### Inferring Single-Cell Fluxes via pH Landscape Modeling

Measurements performed on the cell culture at any given time point yield (a) values of the pH (negative log-concentration of protons) at *M* locations and (b) the positions of *N* cells in a square region of linear size *L* = 500 μm. Given (a) and (b), we want to determine the net proton exchange flux (import or export) for each cell. We solved this problem under a few simplifying assumptions. First, the proton concentration profile is taken to be stationary over our sample. This choice is motivated by the fact that experimental time scales (minutes) are much longer than the time scales over which concentrations are expected to equilibrate (seconds, assuming a diffusion coefficient *D* ≃ 7 × 10^3^ μm^2^ s^–1^^62^) in regions of size *L*. In turn, stationarity implies that concentration profiles solve the Laplace equation ∇^2^*c*(**r**) = 0. Our second assumption is that the solution of the Laplace equation, i.e., the proton concentration at position **r**, can be written as
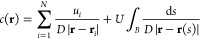
1where **r**_*i*_ denotes the position of cell *i* (*i* = 1, ..., *N*) and the parameters *u*_*i*_ represent the net single-cell fluxes we want to infer. The second term on the right-hand side models the flux *U* from the boundary *B* of the observed frame, whose value is to be inferred along with the *u*_*i*_.

Equation 1 corresponds to the first terms in a multipole expansion of the solution of Laplace’s equation.^63^ The truncation is justified as long as one is interested in the net exchange of protons with the medium by each cell. Higher-order terms in the expansion allow in principle for more refined descriptions (e.g., including separate import and export fluxes, shuttling of molecules inside cells, etc.). However, the larger number of parameters to be estimated would require a much more intensive sampling of the pH profile. By taking eq 1, we effectively focus on the metabolic generation and/or consumption of acids and on the ensuing symport of hydrogen ions. The inverse scaling with distances can be seen as a straightforward consequence of Gauss’s theorem.

We will now denote by *c*_μ_ ≡ *c*(**r**_μ_) the proton concentration measured by the probe at position **r**_μ_ (μ = 1, ..., *M*) and define the matrix of inverse distances . To simplify the notation, we include in the flux vector and in the distance matrix the terms due to the boundary of the frame, i.e., *u*_0_ = *U* and . We can thus define for each frame *t* a cost function
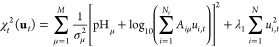
2where, in the first term on the right-hand side, pH_μ_ = −log_10_*c*_μ_ denotes the pH level measured by the probe at position **r**_μ_, while σ_μ_ represents the corresponding experimental error. The second term (λ_1_∑_*i* = 1_^*N*^ *u*_*i*_^2^), which enforces a Gaussian prior for the parameters *u*_*i*_, effectively fixes a uniform scale for exchange fluxes (in agreement with the fact that metabolic fluxes are limited by physical constraints like intracellular and membrane crowding). Terms of this kind are known as Tikhonov regularizers and are widely used in inference problems to prevent multicollinearity.^64^

In order to avoid spurious effects in the inference due to variability in the number of cells in the frame during the experiment (where cells die, divide and migrate, both in and out of the observed frame), we finally consider a total cost function composed by the sum of the cost functions of each frame plus two additional cost terms:
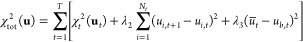
3

The first one, weighted by a parameter λ_2_, is a Tikhonov regularizer ensuring that, for each cell, the change in flux across frames (i.e., at consecutive time points) will not be too large, a reasonable request in view of the fact that the local pH landscape varies on average by no more than 10% over the observation time. The second one, weighted by a parameter λ_3_, constrains instead the overall mean flux  to remain close to the measured bulk value *u*_*b*_ at each time point *t*. We will require that this weighted sum of residuals (the difference between empirical and reconstructed proton levels) is as small as possible. This is equivalent to assuming Gaussian-distributed residuals with inferred fluxes being maximum-likelihood estimates. Confidence intervals and errors on fluxes can be estimated accordingly from the inferred posterior. The minimization of χ_tot_^2^(**u**) was carried out through an optimized Markov chain Montecarlo method (see Methods and Supporting Information for details). Our inference scheme ultimately depends on the parameters λ_1_, λ_2_, and λ_3_, enforcing priors, respectively, for the intensity of single-cell flux, its change in time, and its average bulk value. Their estimate is described in the Supporting Information.

**Figure 3:**
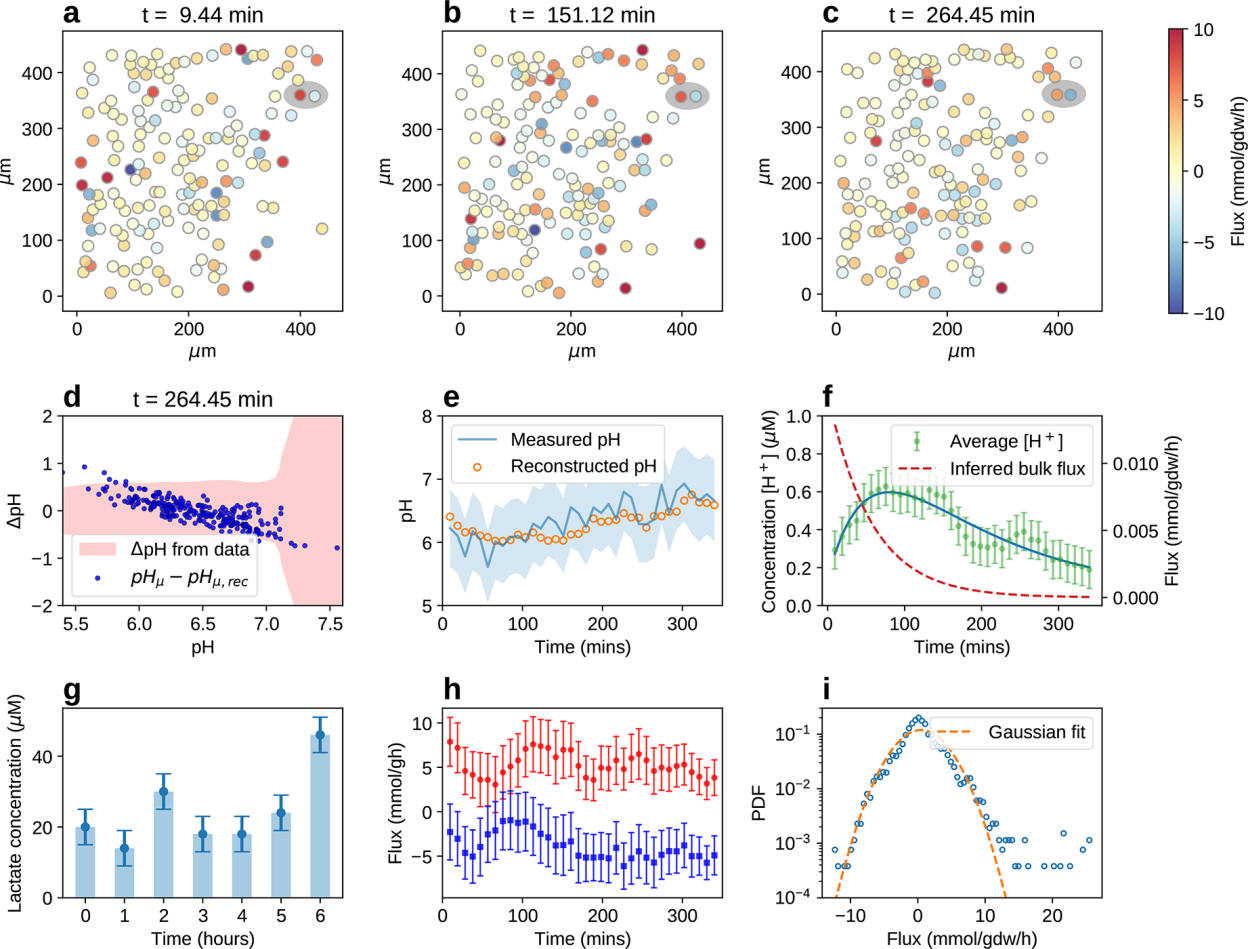
(a–c) Snapshots at different time points (at *t*_*A*_ = 9 min, *t*_*B*_ = 151 min, and *t*_*c*_ = 264 min after the cell culture is settled, all frames are reported in the Supporting Information Figures S1–S5) of the same square visual field (length *L* = 500 μm) during a typical experiment. Cells are represented schematically as disks of diameter 10 μm whose color intensity scales with the flux (side bar, blue vs red for importing vs exporting flux). Probes not shown. (d,e) Quality of the reconstructed pH gradient profile. In (d), the error between the pH calculated from the inferred fluxes and the experimentally observed pH is plotted against the latter for each probe (at time *t*_*c*_ = 264 min, all frames are reported in the Supporting Information Figures S6–S10). In (e), the time trace of the pH measured by a given probe is reported alongside the reconstructed trend at that spatial point. Shaded areas represent the experimental error on the pH at the probes. (f) Time trends of the bulk [H^+^] concentration (experimental, dots and reconstructed, continuous line, left *y* scale) and inferred bulk acidic efflux (dashed line, right *y* scale). (g) Time trend of the experimentally measured bulk lactate concentration in a biological replicate. (h) Single-cell flux intensity (in mmol/gdw/h) as a function of time (in min, sampling every 10 min) of the cells forming the dipole motif highlighted in the upper right corner of the frames in (a–c). (i) Single-cell experimental flux distribution (in mmol/gdw/h, (dots) and its Gaussian approximation (lines) in linear-logarithmic scale. The histogram is built from all single-cell flux values (100–200 cells per frame) and time frames (36 frames resulting from a 6 h experiment sampled every 10 min) tracked in one visual field of one experiment.

Figure 3 displays a typical result from our experiments. Figure 3a,b,c shows the single-cell fluxes (color scale in mmol/gdw/h; the flux unit has been chosen to facilitate comparison with typical bulk values and assuming an average cell dry weight of 0.5 ng); cells are schematically represented as disks of diameter 10 μm in the same visual field at three different time-steps after cell deposition. Single-cell fluxes appear to be evenly distributed in sign, i.e. roughly half of the cells secrete protons while the other half imports them. The quality of the reconstruction is illustrated in Figure 3d,e in space and time, respectively. In Figure 3d, the error between the reconstructed pH at the probes and the observed value is scattered against the observed value (their difference being the residual). More than 90% of the errors lies within the measured standard deviation (shades), although we detect a slight systematic deviation at low and high pH, that is however still within two standard deviations and amounts effectively to a smoothening of the gradient. In Figure 3e, the comparison between reconstructed and measured pH in time is drawn for a given probe. In Figure 3f the bulk behavior of the pH (experimental, dots, and reconstructed, continuous lines) and the inferred bulk efflux (dashed line) are reported, while Figure 3g displays the measured lactate levels in time that quantitatively correlate with the integral of the bulk efflux. The bulk behavior is in good agreement with previous experimental findings,^65^ but single-cell flux values are much higher. The bulk acidification observed during the Warburg effect therefore can be interpreted to be a mere leakage spilling from an intense network of cellular exchanges. In quantitative analogy with electrostatics, we highlight the presence of dipolar interaction motifs. The intensity of one motif as a function of time is shown in Figure 3h: the flux values of dipole-forming cells are mutually correlated and exceed the bulk value by 3 orders of magnitude (10 mmol/gdw/h vs 10^–2^ mmol/gdw/h). A general feature of the set of measured single-cell fermentation fluxes is its strong heterogeneity. This aspect has been analyzed by building the empirical distribution shown in Figure 3g. One sees that the tails strongly deviate from the superimposed Gaussian fit. This quantifies the intuition that a handful of cells carrying extreme fluxes are indeed responsible for a macroscopic fraction of the acidification level in the observed visual field.

### Reconstruction of the Cell-to-Cell Exchange Network

In order to recast single-cell fluxes as pairwise exchange connections, we observe that, in general, given a particle that starts to diffuse at **r**_0_ and *A* absorbing points at positions **r**_*j*_ (*j* = 1, ..., *A*), the probability that the particle is absorbed by one of them also satisfies the Laplace equation.^66^ This means that, if *P*(**r**_*j*_|**r**_0_) denotes the probability that the particle initially at **r**_0_ is absorbed at **r**_*j*_, then
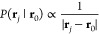
4Based on this, we define the flux from cell *i* (with *u*_*i*_ > 0) to cell *j* (with *u*_*j*_ < 0) as
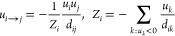
5where *d*_*ij*_ = |**r**_*j*_ – **r**_*i*_|. The term  links the exchange between *i* and *j* to the magnitude of their proton fluxes and to how far *i* and *j* are from each other (the farther away they are, the less likely it is that they are connected as per eq 4). The normalization factor *Z*_*i*_ simply ensures that
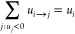
6This defines a weighted directed network for our system that we can easily compute and analyze (see the Supporting Information for further details).

**Figure 4:**
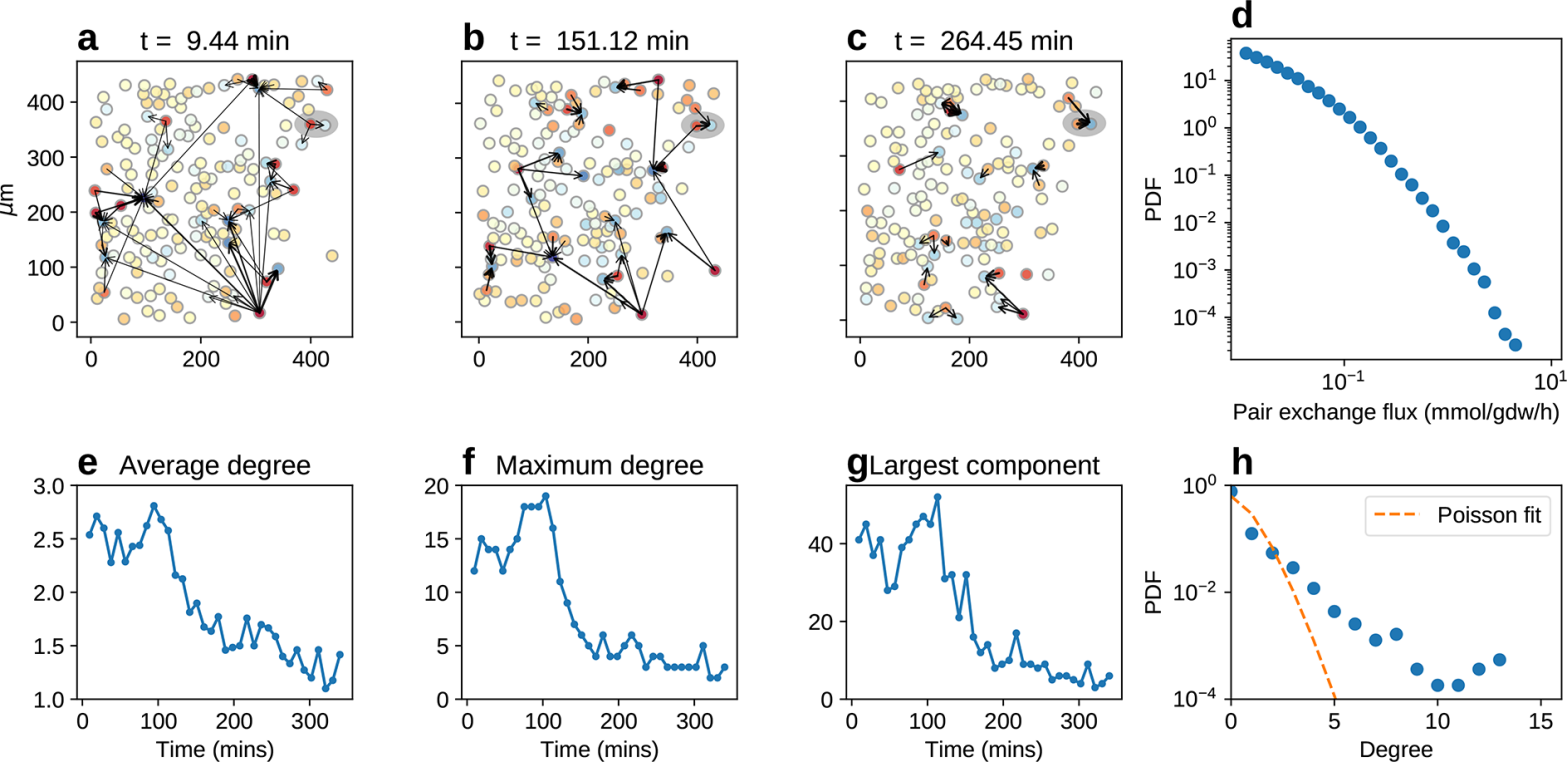
(a–c) Same snapshots of Figure 3a–c with superimposed inferred network structures. Arrows are drawn if the pairwise exchange flux exceeds 0.5 mmol/gdw/h, with a thickness proportional to flux intensity. (d) Distribution of pairwise exchange fluxes (in mmol/gdw/h) in double logarithmic scale (sampled over all frames). (e–g) Structural features quantifying the topology of the flux exchange network as a function of time (in min): average degree (e); degree of the node with maximum connectivity (f); and size of the largest connected component (g). (h) Degree distribution over all frames (dots) and corresponding Poissonian null hypothesis (same mean, lines).

Figure 4a–c showcases the same three snapshot of the system displayed in Figure 3a–c, with the aforementioned network structure superimposed. Arrows are added between cells whose exchange exceeds our average sensitivity 0.5 mmol/gdw/h. The heterogeneity of fermentative phenotypes highlighted in Figure 3e,f is at the origin of the strong heterogeneities in the intensities of exchange fluxes, whose empirical distribution is reported in Figure 4d. One indeed sees that it spans 3 orders of magnitude.

We finally performed a standard graph theory analysis, in particular to calculate the time dependence of the average degree , of the maximum degree, and the size of the largest connected component. These quantities are reported respectively in Figure 4e,f,g. Upon comparing Figure 4a and 4c, one sees that the trends highlight a crossover between qualitatively different regimes. At shorter times, a whole-frame-spanning exchange network is present, sustained by “hub” cells carrying high intensity fluxes. At longer times, such a network appears to dissolve, leading to a phase dominated by isolated dipoles. The topological structure develops in correlated fashion with the bulk pH acidification (Figure 3f). The presence of hubs can be appreciated upon looking to the experimental degree distribution depicted in Figure 4h as compared with a Poissonian null hypothesis of the same mean. The general trend is not significantly perturbed upon varying the threshold defining the links, that is the control parameter for the average network connectivity.

## Conclusions

In this work, we proposed and tested a method for the measurement of single-cell fermentation fluxes, based on high spatial resolution cell seeded pH sensor scaffolds and constraint-based statistical inference. The inverse character of the methodology makes it noninvasive and we applied it to follow in real time the acidification of the environment surrounding a cancerous population (Warburg effect) at the resolution of single cells, also in complex cellular systems, such as tumor and stromal cell cocultures. In short, we highlighted the existence of a network of proton exchanges among cells in line with the lactate shuttle hypothesis and quantified its topology and time evolution over the experimental time scales.

One of the most straightforward application of our method would be thus to probe the lactate shuttle hypothesis in physiological and pathological contexts, like the neuron–astrocyte and tumor–stroma metabolic partnership. The quantification of the exchange intensity reveals strong heterogeneity where a handful of cells is responsible for a large fraction of the pH gradient. Extreme single-cell flux values are of the order of 10 mmol/gdw/h and overcome bulk values (roughly 10^–2^ mmol/gdw/h) by at least 2 orders of magnitude (Figure 3a–c,f). The former values are compatible with those measured in microbic overflows^67^ while the latter are consistent with previous measurements of the (bulk) Warburg effect.^65^

From a biochemical perspective, such fluxes potentially include contributions from a number of different processes (some of which have been detailed in the Introduction) that effectively modulate extracellular pH. The aggregate result of these highly heterogeneous exchanges at population level is however a modest proton leakage flux that we can qualitatively correlate to the increase in lactate levels in culture (see Figure 3g). In other terms, single-cell processes other than lactate secretion appear to roughly balance out at the aggregate level. This confirms the idea that acidification in cancer cell cultures is mainly driven by the accumulation of fermentation byproducts. From our analysis we are however unable to distinguish quantitatively at the level of single cells how other processes contribute to proton exchange fluxes. A direct appraisal of the role of other mechanisms would require genetic benchmarks for the expression levels of specific proton pumps. Alternatively, a more refined model of the metabolic network of the cells might be employed in the inference scheme, leading to an indirect estimate of such quantities.

We further point out that we do not detect any difference in the flux distributions conditioned on the cell type (stromal vs tumoral) and therefore we cannot provide any support to theories based on the idea that different cell types consistently play different roles (and rely on different substrates) in the exchange network (see the Supporting Information for further details). In this respect, our results seem to reject the scenario, suggested by the parallel with carbon overflow in bacteria, according to which fast-growing cancer cells are proton donors and slow-growing fibroblasts are proton acceptors. It is not clear that such a “division of labor” exists in mammalian systems. Recent experimental evidence actually seems to go in the opposite direction.^68^ A similar situation has also been proposed in the context of brain energy metabolism, where high rates of neurotransmission appear to be sustained by an exchange network involving high-ATP-consuming neurons and low-ATP-consuming glia.^69^

To further uncover the mechanism at work behind the Warburg effect and overflow metabolism, it would be important to correlate the measurements of single-cell fermentation with putative determinants of the overflow, like for instance single-cell growth and/or oxidative rates. This would in principle allow to resolve both the whole carbon flux at single-cell resolution and the ensuing intercellular interactions. Such measurements would impact on our understanding of the ecology and metabolism of cellular populations, providing the experimental ground for recent quantitative theoretical approaches based on statistical mechanics.^70−78^

## Methods

### Chemicals

Polycaprolactone (PCL, molecular weight 80 000 g mol^–1^, 440744, Sigma-Merck, KGaA, Darmstadt, Germany), chloroform (puriss. P.a., reag. ISO, reag. Ph.Eur., 99.0–99.4% (GC) 32211, Sigma-Merck, KGaA, Darmstadt, Germany), DMSO (dimethyl sulfoxide, BioUltra 99.5% GC 41639, Sigma-Merck, KGaA, Darmstadt, Germany) and ethanol (puriss. p.a., ACS reagent, Reag. Ph.Eur., 96% v/v 32294, Honeywell, USA) were used for the fabrication of electrospun fibers. Tetraethyl orthosilicate (TEOS, item code: 131903), (3-aminopropyl)triethoxysilane (APTES, item code: 440140), potassium chloride (item code: P9541), ammonium hydroxide solution 28% (item code: 211228), fluorescein 5(6)-isothiocyanate (FITC, item code: 46950), Rhodamine B isothiocyanate (RBITC, item code: R1755), and tygon formula 2375 laboratory tubing i.d. × o.d. 1.6 mm × 3.2 mm (item code: Z685585) were purchased from Sigma. A 50 mL syringe was purchased from HSW HENKE-JECT, NE-4000 syringe pump from New Era pump systems.

### Cell Lines

Human pancreatic cancer cell line AsPC-1 (ATCC CRL-1682) was obtained from American Type Culture Collection (ATCC, Rockville, Md., USA) and cultured at 37 °C in a humidified 5% CO_2_ incubator according to ATCC protocols. Cancer-associated fibroblasts, CAFs (Vitro Biopharma, cat. no. CAF08), were cultured in DMEM (D5671, Sigma-Merck KGaA, Darmstadt, Germany) supplemented with 10% FBS (F7524, Gibco, Thermo Fisher Scientific), 2 mM glutamine (G7513, Sigma-Merck KGaA, Darmstadt, Germany), 1% penicillin/streptomycin (P0781, Sigma-Merck KGaA, Darmstadt, Germany), and 1 μg/mL recombinant human fibroblast growth factor-basic (FGFb) (catalog #13256-029, Gibco, Thermo Fisher Scientific) at 37 °C with 5% CO_2_. Cell lines were subcultivated with a ratio of 1:5 and passaged 2 times per week. Mycoplasma contamination was routinely tested by a mycoplasma PCR detection kit (G238 abmGood, Canada).

### Image Analysis

Input data are raw images which, concretely, consist of information collected by three independent channels:

- Red Channel (particles emit a constant signal regardless of local pH)
- Green Channel (particles emit a signal proportional to local pH)
- Blue Channel (this is related solely to nuclei emission; hence it helps splitting cells from sensors)

Two algorithms were applied in series to all images as preprocessing steps, the former [Algorithm A] to identify cells or sensors within the image, and the latter [Algorithm B] to quantify their intensity and hence their pH read-outs (for sensors only). More details are reported in the Supporting Information.

### Monte Carlo Method

The inference setup described above is a maximum likelihood problem where fluxes are assumed to be distributed as *P*(**u**) ∝ exp [−χ_tot_^2^(**u**)], and the optimal guesses for **u** are those that maximize the probability of occurrence. Points of the posterior distribution have been sampled with an optimized Monte Carlo method based on the Metropolis-Hastings algorithm,^79^ i.e., by defining a Markov chain in the flux space based on a random walk whose steps have conditional probabilities , where  is the variation of log-likelihood  in going from **u** to **u**′. An analytical Gaussian approximation of the log-likelihood rate function has been used to over-relax the random walk to tackle ill-conditioning and provide a warm start. The maximum likelihood optimal configuration has been found through simulated annealing while confidence intervals and errors have been estimated numerically by exploiting the invariance properties of the log-likelihood functions. More details are reported in the Supporting Information while codes implementing the method are available at https://github.com/demartid/infer_single_cell_fermentation_codes_data.

## Supporting Information Available

Details about the experimental and computational methods, including the characterization of the pH sensing nanofibers (S1, S2, S4), the pH calibration curve (S3), reconstructed pH maps (S8), and flux distributions conditioned to the cell type (S7) (PDF)

## Notes

This article was deposited on a preprint site May 4 2022. Onesto, V.; Forciniti, S.; Alemanno, F.; Narayanankutty, K.; Chandra, A.; Prasad, S.; Azzariti, A.; Gigli, G.; Barra, A.; De Martino, A.; De Martino, D.; del Mercato, L.L. Probing single cell fermentation flux and intercellular exchange networks via pH-microenvironment sensing and inverse modeling. *bioRxiv***2022**; 10.1101/2022.05.03.490288.
