# Supplementary material for: Probing Single-Cell Fermentation Fluxes and Exchange Networks via pH-Sensing Hybrid Nanofibers
Source: ACS Nano. 2022 Dec 27;17(4):3313–23. doi: 10.1021/acsnano.2c06114 (PMC9979640; doi:10.1021/acsnano.2c06114)
Supplement: Supplementary file 1 — nn2c06114_si_001.pdf [file nn2c06114_si_001.pdf]

Supporting Information: Probing single cell fermentation fluxes and exchange networks via pH-sensing hybrid nanofibers

Valentina Onesto,† Stefania Forciniti,† Francesco Alemanno,†,‡,¶ Krishnadev Narayanankutty,§ Anil Chandra,† Saumya Prasad,† Amalia Azzariti,‖ Giuseppe Gigli,†,‡ Adriano Barra,‡,¶ Andrea De Martino,⊥,# Daniele De Martino,*,§,@ and Loretta L. del Mercato*,†

†*Institute of Nanotechnology, National Research Council (CNR-NANOTEC), c/o Campus Ecotekne, via Monteroni, 73100, Lecce, Italy.*

‡*Dipartimento di Matematica e Fisica E. De Giorgi, University of Salento, 73100, Lecce, Italy.*

¶*Istituto Nazionale di Fisica Nucleare (INFN), Sezione di Lecce, 73100, Lecce, Italy.*

§*Biofisika Institutua (UPV/EHU, CSIC) and Fundación Biofísica Bizkaia, Leioa E-48940, Spain.*

‖*IRCCS Istituto Tumori Giovanni Paolo II, V.le O. Flacco, 65, 70124, Bari, Italy.*

⊥*Politecnico di Torino, Corso Duca degli Abruzzi, 24, I-10129, Torino, Italy.*

#*Italian Institute for Genomic Medicine, IRCCS Candiolo, SP-142, I-10060 Candiolo (TO), Italy.*

@*Ikerbasque Foundation, Bilbao 48013, Spain.*

E-mail: daniele.demartino@ehu.eus; loretta.delmercato@nanotec.cnr.it

Further details on experimental methods

Synthesis of particle-based pH-sensors

Ratiometric fluorescent pH-sensors based on silica microparticles were synthesized via recent work.[S1] Briefly, dye molecules of FITC and RBITC were covalently linked with APTES in ethanolic solution for 3 hours in dark, 5 mg FITC and 6.5 mg RBITC were dissolved respectively in 3 mL anhydrous ethanol followed by addition of 13 µL APTES (20.6 mM). The two solutions of respective dyes with APTES were kept on magnetic stirrers for 4 hours at room temperature in dark. The formed FITC-APTES and RBITC-APTES conjugates were used directly in the next step without further purification. In the next step the silica microparticle buildup starts with the formation of seed suspension (Step 1) followed by its growth (Step 2) by slow addition of the monomer TEOS and dye-APTES conjugated molecules. The seed formation is achieved by dissolving 23 mg KCl in 9.45 mL of deionized water in a round bottom flask followed by addition of 96 mL of absolute ethanol, 6 mL of ammonium hydroxide (28%) and 1.73 mL of TEOS. The solution in the flask was continuously stirred using a magnetic bead at 240 rpm for 30 minutes. The next step involved increasing the size of the seed particles by slowly adding monomer TEOS along with dye conjugated APTES. Here 4.4 mL of TEOS, 2 mL of FITC-APTES and 2 mL of RBITC-APTES were dissolved in 33 mL of absolute ethanol. This solution was then slowly added drop by drop (flow rate: 0.05 mL/min) into the seed solution that is being stirred at 240 rpm, using a 50 mL plastic syringe and tubing. During the seed formation and growth of the fluorescent silica particles the flask was kept airtight to using septum to prevent ammonia leakage. The reaction proceeded for 24 hours followed by careful pipetting of the supernatant, leaving larger debris of aggregated particles in the bottom. The collected particles in the supernatant were washed three times in ethanol using centrifugation at 2000 rpm followed by washing with deionized water thrice. The particles were stored in ethanolic suspension at 4 °C.

Fabrication of pH-sensing hybrid nanofibers

PCL solution for electrospinning was prepared using chloroform/DMSO in 90:10 v/v ratio. PCL was first dissolved in 900 µL of chloroform at a concentration of 10% (w/v), at room temperature with overnight stirring. 36 mg of pH-sensing microparticles were dispersed in 100 µL of DMSO, transferred in an ultrasound bath (Digital Ultrasonic Cleaner, Argo Lab) at 20 °C for 30 minutes to reduce aggregation, and finally added to 900 µL of PCL solution in chloroform. After addition of the pH-sensing microparticles, the resulting solution (1 mL) was left stirring for 4 hours at room temperature. 1 mL of the solution was loaded in a 1 mL plastic syringe with 21G stainless steel needle and the syringe was placed on a syringe pump (E-fiber, SKE Advanced Therapies). A horizontal electrospinning set-up was used with a custom rotating collector having a rotating speed of 2200 rpm. The applied positive and negative voltages were +14 kV and −6 kV respectively, the tip of the needle to collector distance was 10 cm and the feeding rate was 0.8 mL/h. The hybrid organic nanofibers were collected on 1 cm × 1 cm glass slides positioned on the target for 30 seconds (for CLSM imaging) and 90 seconds (for SEM imaging). Before electrospinning, the glass slides were cleaned in a bath of ethanol for 5 minutes and dried with compressed nitrogen gas. All the experiments were conducted at room temperature (19 °C-22 °C) and a relative humidity of 35% within a closed chamber.

Characterization of pH-sensing hybrid nanofibers

Morphological characterization of the pH-sensing nanofibers has been carried out by scanning electron microscopy (SEM, Sigma 300VP, Zeiss, Germany) analysis (accelerating voltage of 3 kV , secondary electron detector (SE2), 5000x, 10000x and 30000x magnifications). All the samples were sputter coated with an 8 nm thick gold layer (Safematic CCU-010 LV Vacuum Coating) prior to their observation under the microscope. SEM images were analyzed on ImageJ[S2] software by drawing linear regions of interest at random locations to extract the average fiber diameter. The thickness of the pH-sensing fibrous film was extracted by z-stack

CLSM (LSM 700, Zeiss, Germany) acquisitions (objective 20x/0.8, z-step=0.1 µm) in three different areas (213.39 µm × 213.39 µm) of three different scratched samples (figure S1). The thickness of our system resulted being 2.91 ± 0.17 µm (mean ± SEM) (figure S1).

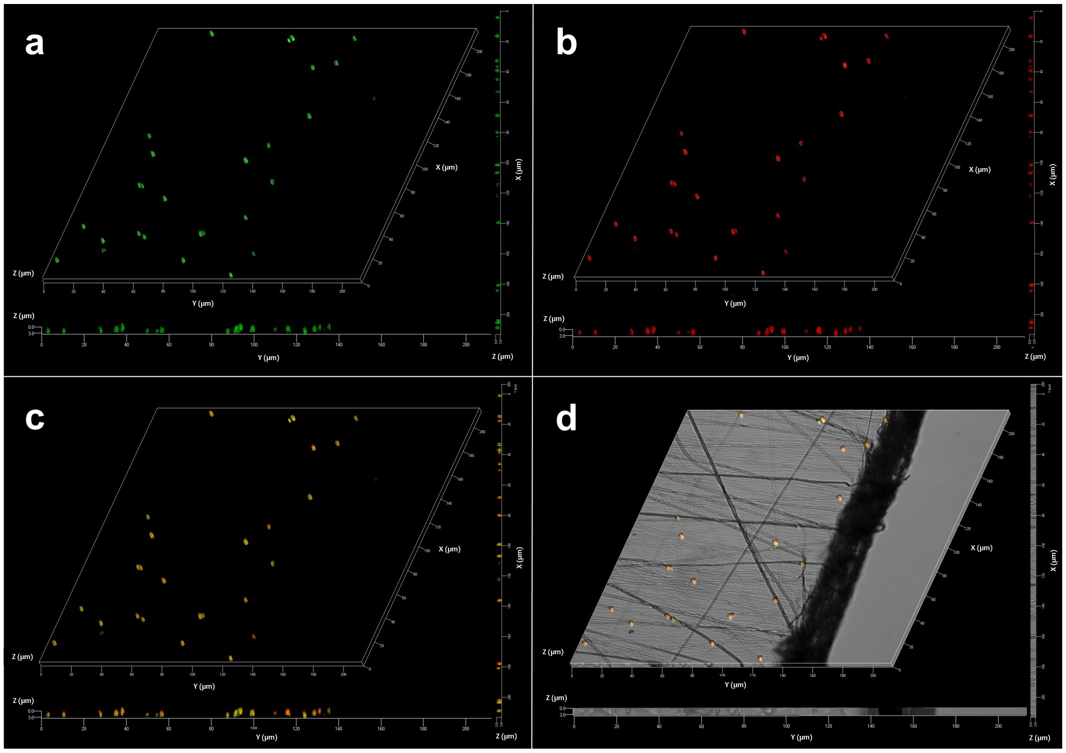

Figure S1: Representative 3D reconstruction and orthogonal projections (z-y on the bottom and z-x on the left of each 3D reconstruction) of CLSM micrographs of a scratched sample of pH-sensing fibers. a) FITC (green channel), b) RBITC (red channel), c) overlay (green+red channels), d) overlay with bright field.

To assess the buffering capacity of the hybrid pH-sensing system a pH titration on a fixed volume (6 mL) of PBS was done with on PBS buffer, PBS with pH sensors and PBS with pH-sensing fibers. To these individual solutions a known volume (5 µL) of 1 N HCl was progressively added and the corresponding pH change was monitored using a pH electrode. The plot profile of the titration is shown in Figure S2.

Assessment of titration data showed that the sensor particles as well as the fibers do not have significant buffering capacity compared to PBS between pH 7.4 and 6.5. However, below pH 6.5, further addition of same acid volume (5 µL) resulted in different pH reductions in the three solution types, where PBS reached lowest pH value of pH ≈5.5, pH sensors dispersed

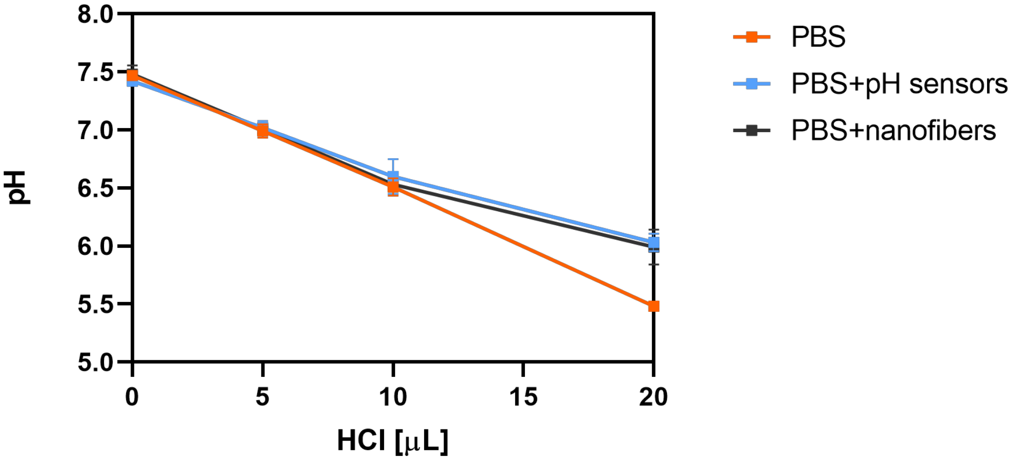

Figure S2: Buffering capacity comparison. pH titration on a fixed volume of PBS on (i) PBS buffer, (ii) PBS with pH sensors (iii) PBS with pH-sensing fibers.

in PBS reached pH ≈6.03 and fibers with sensors dispersed in PBS reached almost same pH value of pH ≈5.99. This variation in the extent of pH decrease can be associated with the additional buffering action of sensor particles and fibers in addition to the PBS. A more detailed calculation showed that 4.85×10^{-19} and 9.10×10^{-18} moles of hydrogen ions get adsorbed on single sensor particles and respective single sensors inside the fibers. Considering the average number of moles of hydrogen ions secreted by a single cancer cell per minute to be 3.47×10^{-14} moles,[S3] the buffering caused by the sensor particles is negligible. In other words, the protons adsorbed by one sensor is just 0.0014% of the protons released by one cell in one minute. Similarly, one sensor particle encapsulated in the fiber can adsorb just 0.0262% of protons released by a cell in one minute. Therefore, to capture all the protons produced by a cell roughly 7.16×10^{4} particles would be required around that cell. As the number of sensor particles around cells in our experiment is much less, there is negligible buffering action imparted by the sensors. Thus, we believe that the measurement of pH by the sensors will not alter the system under study and the pH values read outs reflect the true pH in the cellular microenvironment.

Calibration of pH-sensing hybrid nanofibers

Calibration of pH-sensing matrices was performed by adding 200 µL of pH-adjusted cell medium (pH values 5.0, 6.0, 7.0, 8.0) on the top of the nanofibers. Samples were allowed to equilibrate for 15 minutes, and then imaged using a confocal laser-scanning microscope (CLSM, SP8 Leica Microsystem, Mannheim, Germany) at $\lambda_{exc} = 488\,\text{nm}$ to collect the fluorescence signals in the green channel ($\lambda_{em} = 500 - 550\,\text{nm}$) and at $\lambda_{exc} = 561\,\text{nm}$ to collect the fluorescence signals in the red channel ($\lambda_{em} = 570 - 620\,\text{nm}$). At least four CLSM images (232.5 µm × 232.5 µm) were acquired for each pH point, with around 300 sensors in each image. Images were then processed as described in section "Further details on image analysis". The equation of the fit calibration curve (Figure S3) was used to estimate unknown pH experienced by the sensors in a region of interest.

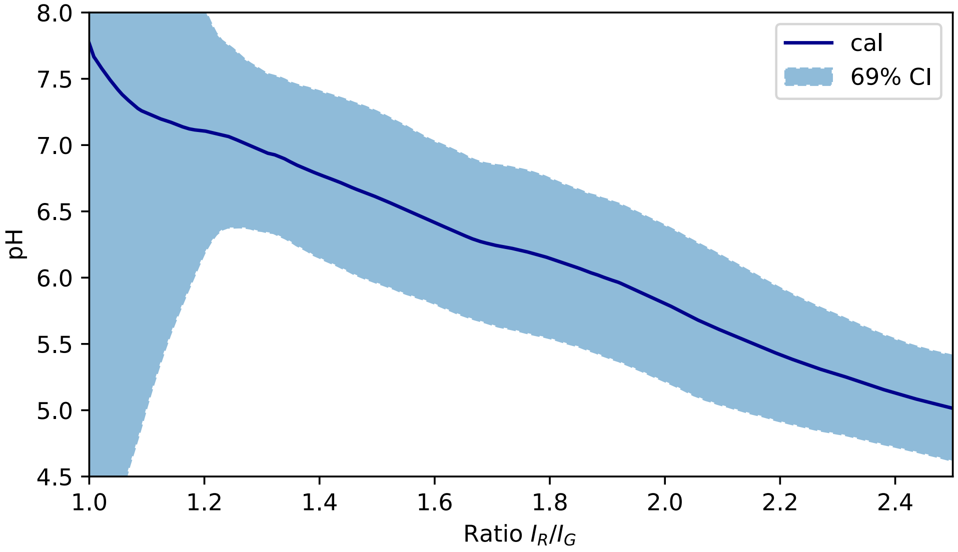

Figure S3: Calibration of pH-sensing nanofibers. Ratiometric calibration curve of the pH-sensing nanofibers. Shaded area represents a 69% confidence interval (CI) on the pH values (i.e., at a fixed I_R/I_G ratio, 69% of the observed pH values will fall within the shaded area).

Cell proliferation assay

A suspension of 15000 cells/well were seeded in the wells of a 24-well cell plate and on PCL-sensing fibers. Cell proliferation was measured at days 1, 4 and 8 through the CyQuant Cell proliferation assay kit (Invitrogen, Thermo Fisher Scientific). Briefly, at each time point the medium was removed from the wells. After washing in PBS 1X, cells were frozen at $-80\,°C$ for at least 3 hours. Then, the plates were thawed at room temperature and 200 µL of the CyQUANT® GR dye/cell-lysis buffer were added in each sample well and incubated for 5 minutes. The fluorescence signal of the GR-dye, which bound to cellular nucleic acids, was measured using a microplate reader (ClarioStarPlus, BMG Labtech) with excitation at 485 ± 10 nm and emission detection at 530.0 ± 12.5 nm. Cells grown directly in the well plate were used as a control.

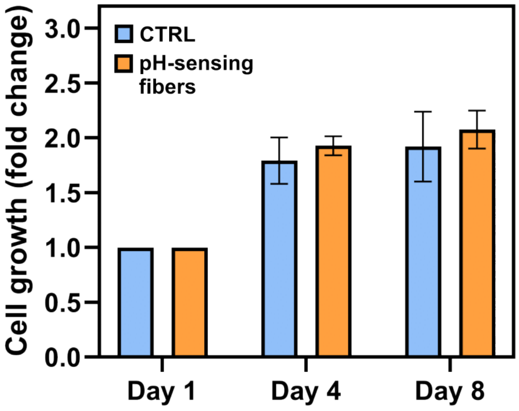

Figure S4: Cell viability of AsPC-1 and CAFs co-cultured on pH-sensing nanofibers. Histograms display the fluorescence measurements of the GR-dye, which bound to cellular nucleic acids, at 1, 4 and 8 days from cell seeding. Values are the means (±SE) of three independent experiments.

Cell co-cultures on pH-sensing hybrid nanofibers

Prior to the cell culture experiments, the pH-sensing nanofibers deposited onto 1 cm × 1 cm glass slides were sterilized by UV exposure for 30 minutes and placed into μ-slide 4 well chamber slide (IBIDI GmbH, Grafelfing). AsPC-1 tumor cells and CAFs were counted by

Trypan Blue dye exclusion and 4×10^4 cells were seeded in each well in the ratio 70% CAFs and 30% AsPC-1 tumor cells. In order to facilitate cell tracking by automated algorithms, both cell types were stained with a nuclear marker, Hoechst 33342 (B2261, Sigma Aldrich) 1:1000, and only tumor cells were stained with a plasma membrane marker, CellMask TM Deep Red (C10046, Invitrogen, ThermoFisher Scientific) 1:1000 for 20 minutes. After washing in PBS 1X, cell suspension was pipetted drop by drop on the pH-sensing matrices and then incubated over night at 37 °C with 5% CO_2 to allow cell attachment.

Extracellular lactate quantification

Changes in lactate concentration in cell culture medium were measured using the Lactate-Glo™ Assay Kit (Promega® Madison, WI, USA) according to the manufacturer's instructions. Briefly, AsPC1 tumor cells and CAFs were seeded on pH-sensing nanofibers as described in section "Cell co-cultures on pH-sensing hybrid nanofibers". After cell attachment, the medium was changed in each well and left for 15 minutes to equilibrate. Each sample was collected every hour by diluting 5 µL medium into 95 µL of PBS. Cell culture medium collected from each experimental time point were then subjected to an enzymatic reaction coupling lactate oxidation and NADH production with a bioluminescent NADH detection system provided by the Lactate-GloTM Assay kit. Luminescence was recorded using a microplate reader (ClarioStarPlus, BMG Labtech). Medium without cells was used as negative control for determining assay background. The data generated were normalized by dividing the relative luminescence units (RLU) by the cell number obtained for each condition. Lactate concentration was calculated through a standard curve using a titration of lactate and all measured samples were within the linear range from 0 to 200 µM. Data represent the average (±SEM) of three independent experiments.

Statistical significance

Data extracted from time-lapse experiments are the mean ± standard error of the mean (SEM) of three independent experiments. Three SEM images were acquired for each magnification (5000x, 10000x and 30000x) from three different samples. Fiber diameters extracted from the SEM images are presented as mean ± SEM of 100 individual measurements from each sample. Each time-lapse experiment was compared with the proper calibration curve to convert the fluorescence value to a precise pH value.

Sensing of pH in the cell cultures over time

pH variations of cells cultured on pH-sensing nanofibers were measured over time for 6 hours with a time interval of 10 minutes. Before each time-lapse acquisition, calibration of the system composed of cells on pH-sensing matrices was performed by adding 200 µL of pH-adjusted Leibovitz L15 medium (cat. num 21083027, Gibco, Thermo fisher Scientific). Samples were allowed to equilibrate for 15 min, and then imaged by a CLSM Leica SP8 with a HC PL FLUOTAR 20x/0.50 (0.5NA) objective, maintaining a temperature of 37 °C. 200 µL of L15 medium were added to the sample composed of cell co-cultures on pH-sensing matrices before starting pH monitoring. During time-lapse CLSM measurements, FITC was excited with $\lambda_{exc} = 488\,nm$ ($\lambda_{em} = 500 - 550\,nm$), RBITC was excited with $\lambda_{exc} = 561\,nm$ ($\lambda_{em} = 570 - 620\,nm$), Hoechst was excited with $\lambda_{exc} = 405\,nm$ ($\lambda_{em} = 415 - 500\,nm$), Deep Red was excited with $\lambda_{exc} = 633\,nm$ ($\lambda_{em} = 640 - 750\,nm$). All images were acquired for a $232.5\,\mu m \times 232.5\,\mu m$ field at 696×696 pixel resolution (zoom factor 2.5) using a pinhole setting of 1 airy unit and collecting a z-stack of ~ 19 slices (step size: 1.55 µm). To image the whole sample, for each time point, 2×2 tile scans were acquired and stitched into larger mosaics.

Further details on image analysis

[A] Particle (cell or sensor) detection

Assuming particles are brighter than the background, at first we store the maximal intensity level of the image (that we call M), then we apply a spatial band-pass filter (BPF) with the band passing through the pixels $[R/2, 3R/2]$, R being the candidate radius of the particle and the bandwidth, obtaining a filtered image FI, then we further apply a dilatation[S4] to the filtered image to obtain a second filtered image (DFI). Points accounting for centers of the particles then fulfill the conditions

$$(\mathrm{DFI} = \mathrm{FI}) \quad \text{and} \quad (\mathrm{DFI} > \mathrm{M} \cdot \mathrm{Tol}) \tag{1}$$

where the parameter Tol (tolerance) ranges in (0,1): the first condition ensures that the pixel is a local maximum, the second condition guarantees that the luminosity of the candidate center is brighter enough for further processing.[S5]

[B] Intensity Evaluation

To estimate the average luminosity I of each point, we calculate an integral over the radius of the particle using the formula

$$\bar{I} = \exp\left[\frac{\sum_p I(p)\log I(p)}{\sum_p I(p)}\right] \tag{2}$$

where $I(p)$ is the intensity of the given pixel p. To track sensors procedure [A] is applied to the sum of the Green and Red channels in order to obtain the positions of all the particles within the image. Using as center of integration, time by time, all the candidate center positions previously detected, procedure [B] is then applied separately to the red and green channels, obtaining, for each particle, the ratio R of their intensities $R = I(\text{red})/I(\text{green})$.

This ratio is then transformed, using the calibration curve, to a pH value. To track cells instead, the sole usage of procedure [A] suffices.

[C] Tracking cells and probes across frames

The recorded positions of individual cells and probes vary across time not only due to the motion of cells but also due to the lateral shifts during the imaging process. For tracking objects across time, we employed a simple method based on comparing the positions of individual objects across consecutive time points and finding the closest match: Particle i of a time point and particle j of another time point are considered to be the same particle if the separation d_{ij} between them is a minimum w.r.t. both variation of i as well as that of j. This method was considered sufficient since the maximum displacement of the objects, as observed from the images, across any one interval in time was smaller than the minimum separation between the objects in any single frame. There were instances of cells at the boundary leaving and re-entering the frame of imaging, and also cells crossing over each other during motion, at which point our algorithm fails to track these cells anymore. In case of probes, the tracking fails when the lateral shifts during imaging are comparable to the minimum separation between individual probes, and also when cells pass over any probes affecting the imaging of those probes. With this rather conservative algorithm, we were able to successfully track more than 65% of the cells and more than 80% of the probes.

Further details on computational methods

Gaussian approximation

We will describe here an analytically tractable approximation of the inference task. This will play a fundamental role for the Monte Carlo Markov chain to solve the full non-linear problem, in order to

1. Provide a warm start: the approximate maximum likelihood point will be taken as initial point for the sampling thus reducing the transient equilibration time.

2. Tackle ill-conditioning: the approximate covariance matrix will be used to define a coordinate transformation (or in practice preferential sampling direction) that rounds the space and reduce sampling times by the condition number, i.e. the ratio between the maximum and minimum eigenvalues.

For sake of simplicity in notation we will consider here the expression for one time frame, that can be easily generalized to the full time evolution. Let us consider the full non-linear likelihood function (for sake of simplicity without priors)

$$\chi^2_{\mathrm{NL}}(\mathbf{u}) = \sum_{\mu} \frac{\left(\mathrm{pH}_\mu + \log_{10}\left(\sum_i A_{i\mu} u_i\right)\right)^2}{2\sigma_\mu^2} \tag{3}$$

Upon considering $c_\mu = 10^{-\mathrm{pH}_\mu}$, we can approximate the residuals

$$\mathrm{pH}_\mu + \log_{10}\left(\sum_i A_{i\mu} u_i\right) = \log_{10}\left(1 + \frac{\sum_i A_{i\mu} u_i - c_\mu}{c_\mu}\right) \sim \frac{\sum_i A_{i\mu} u_i - c_\mu}{c_\mu} \tag{4}$$

that shall be valid for small deviations from the expected value and leads to a Gaussian expression of the likelihood.

$$\chi^2_{\mathrm{L}}(\mathbf{u}) = \sum_{\mu} \frac{\left(c_\mu - \sum_i A_{i\mu} u_i\right)^2}{2 c_\mu^2 \sigma_\mu^2} \tag{5}$$

Notice the weights in agreement with standard error propagation formulae. Standard linear algebra manipulations lead finally to the optimal solution

$$u_j^* = \sum_i (B_{ij})^{-1} b_i \tag{6}$$

$$\text{where} \qquad b_i = \sum_\mu \frac{A_{i\mu}}{c_\mu \sigma_\mu^2} \tag{7}$$

$$\text{and} \qquad B_{ij} = \sum_\mu \frac{A_{i\mu} A_{j\mu}}{c_\mu^2 \sigma_\mu^2}. \tag{8}$$

Upon regarding the χ^2 as the large deviation function for the posterior it is straightforward to obtain an analytical approximate expression for the covariance matrix of fluxes

$$\langle u_i u_j \rangle = (\mathbf{B}^{-1})_{ij} \tag{9}$$

where $\langle \ldots \rangle$ indicates the average. This matrix could be used for errors estimation, but it highly overestimates them. In practice, its principal component analysis gives quantitative insights on the typical scales and it has been used to remove the ill-conditioning in the inference via preferential sampling direction in the Monte Carlo Markov chain.

Enforcing positivity constraints of the reconstructed concentration profile

The gaussian approximation has the drawback that the reconstructed profile is not anymore positive definite (a condition that is verified by construction in the full non-linear case). This can be ameliorated upon enforcing ad-hoc constraints that defines a quadratic convex optimization problem. We can thus impose that the reconstructed values of the concentration at the location of the probes $c_{\mu,\mathrm{rec}} = \sum_i A_{i\mu} u_i$ shall strictly be non-negative. This leads to

the problem

$$\begin{aligned} \text{minimise} \quad & \chi^2_{\mathrm{L}}(\mathbf{u}) \\ \text{such that} \quad & \sum_i A_{\mu i} u_i \geq 0 \end{aligned}$$

This is in the standard form of a convex quadratic program, that we solved using the Goldfarb-Idnani algorithm.[S6]

Priors and Lagrange multipliers

Tikhonov regularizer for the scale λ_1

We considered a regularizer λ_1 setting the single cell flux scale. This is done to prevent multicollinearity and it is equivalent to assume a prior taking into account previous experimental knowledge (e.g. on enzyme kinetics). The introduction of regularizers as quadratic terms in the log-likelihood rate function does not change the form and character of the maximum-likelihood solution as it can bee seen in the following. Consider the simplified form

$$\chi^2_{\lambda_1}(\mathbf{u}) = \sum_\mu \left(c_\mu - \sum_i A_{\mu i} u_i \right)^2 + \lambda_1 \sum_i u_i^2 \tag{10}$$

We have

$$\begin{aligned} \chi^2_\lambda(\mathbf{u}) &= \chi^2(\mathbf{u}) + \lambda \mathbf{u}^2 \\ &= \mathbf{c^T c} - 2(\mathbf{c^T A})\mathbf{u} + \mathbf{u^T}(\mathbf{A^T A} + \lambda \mathbf{I})\mathbf{u} \end{aligned} \tag{11}$$

which on comparison with the previous solution it gives the minima $\mathbf{u}^*$ as

$$\mathbf{u}^* = (\mathbf{A^T A} + \lambda \mathbf{I})^{-1} \mathbf{A^T c} \tag{12}$$

$$= (\mathbf{B} + \lambda \mathbf{I})^{-1} \mathbf{b} \tag{13}$$

We have fixed $\lambda_1 = 2.5 \times 10^{-2}$ (mmol/gdw/h)$^{-2}$, in turn corresponding to a typical flux scale of 20 mmol/gdw/h consistently with known values of the maximum rate $V_{\max}$ of monocarboxylate transporters.[S7]

Time continuity across frames λ_2

As we remarked above the number of cells in the observed visual field varies due to (mainly) migration, proliferation and death, with cells entering and exiting the field view. This can introduce spurious terms in the inference: for instance the flux of cells nearby the boundary in one frame that exit the visual field in the next frame could be artificially reassigned to nearby cells. In order to minimize the effect of such artifacts we imposed time continuity across frame for tracked cells (see above) with a term connecting the log-likelihood rate function of different frames

$$\lambda_2 \sum_{i=1}^{N_t} (u_{i,t+1} - u_{i,t})^2 \tag{14}$$

The value of λ_2 has been set to match the observed time gradient for the probes, that is within 10% of the maximum observed variation or around 1 mmol/gdw/h per time frame (10 min), in turn giving $\lambda_2 = 0.5\,(\text{mmol/gdw/h})^{-2}$.

Matching the bulk trend λ_3

Upon averaging over the probes we have a measurement of the bulk pH $\bar{c}(t)$ as a function of time. The dynamics generating the latter can be essentially split into two basic contributions: i) the average or bulk cell efflux $\bar{u}$, and ii) the medium buffering capacity. Upon inferring the former, this can be used to further constraint our single cell flux measurements upon adding a term

$$\lambda_3 \left(\frac{1}{N} \sum_i u_i - \bar{u} \right)^2 \tag{15}$$

in the log-likelihood rate function. We have thus considered the following model for the dynamics in terms of linear perturbation around a steady state (we drop the bars for simplicity

of notation), with parameters k, c_e describing the medium buffering and k_u, u_s

$$\begin{aligned} \dot{c} &= u - k(c - c_e) & (16) \\ \dot{u} &= -k_u u & (17) \end{aligned}$$

It is interesting to notice a quantitative analogy with electrical circuits. If we take the second derivative of the first equation and substitute with the second we have

$$\ddot{c} + (k + k_u)\dot{c} + kk_u(c - c_{eq}) = 0 \qquad (18)$$

If we consider the map $I = c - c_{eq}$, $k + k_u = R/L$ and $kk_u = 1/LC$ we have the equation

$$\ddot{I} + \frac{R}{L}\dot{I} + \frac{1}{LC}I = 0 \qquad (19)$$

that is exactly the equation for the current in a series RLC circuit with a constant e.m.f. The solution in the so-called overdamped regime (where our parameters are) is a sum of decaying exponentials

$$I = A_1 e^{-\lambda_1 t} + A_2 e^{-\lambda_2 t} \qquad (20)$$

where the constants A_1, A_2 depend on initial conditions while the time constants $1/\lambda_1$ and $1/\lambda_2$ depend on the values of the parameters. If A_1 and A_2 have opposite signs, the above function can give rise to a non-monotonous trend with a peak, which is precisely what we find in our case. This linear system has thus a straightforward analytical solution

$$\begin{aligned} u &= u_0 e^{-k_u t} & (21) \\ c &= (c_0 - c_e) + c_e(1 - e^{-kt}) + \frac{u_0}{k_u - k}(e^{-kt} - e^{-k_u t}) & (22) \end{aligned}$$

and its four parameters has been inferred by a simple local grid search. We obtain $k = 0.42 \pm 0.30\,\mathrm{h}^{-1}$, $k_u = 1.0 \pm 0.2\,\mathrm{h}^{-1}$, $u_0 = 0.015 \pm 0.003$ mmol/gdw/h. These values can be

used to make a estimate for the lactate concentration ($\dot{c}_{lac} \sim u$)

$$c_{lac} \sim u_0/k_u \sim 30\,\mu\text{M} \tag{23}$$

that is in quantitative agreement with the experimentally observed ($\sim$ 20 µM-70 µM). The inferred time trend for $u(t)$ has been then used to constrain the average flux in our inference and standard statistical considerations suggest that the lagrange multiplier shall be be set to $\lambda_3 = N\lambda_1 \sim 4$ (mmol/gdw/h)$^{-2}$, where $N \sim 150$ is the average number of cells per frame.

The Monte Carlo Markov chain

The posterior probability distribution for the fluxes is

$$P(\mathbf{u}) \propto e^{-\chi^2_{\text{NL}}(\mathbf{u})} \tag{24}$$

In order to sample from this distribution we employed the well known Metropolis-Hastings algorithm: starting from an initial configuration $\mathbf{u}_0$, define the series $\mathbf{u}_l$

1. Upon perturbing $\mathbf{u}_l$ propose a new vector $\mathbf{u}_{l+1,p}$, calculate the likelihood variation $\Delta\mathcal{L} = \chi^2_{NL}(\mathbf{u}_{l+1,p}) - \chi^2_{NL}(\mathbf{u}_l)$.
2. Accept the new vector $\mathbf{u}_{l+1} = \mathbf{u}_{l+1,p}$ with probability $\min(1, e^{\Delta\mathcal{L}})$, otherwise keep the old one $\mathbf{u}_{l+1} = \mathbf{u}_l$

The efficiency of such method relies on properly defined proposal steps (e.g. avoiding ill conditioning) and on initial points that are as close as possible to the typical equilibrium ones (warm start). The proposal step has been chosen by performing a random walk over the eigenvectors of the covariance flux matrix in the gaussian approximation and choosing as starting point the maximum likelihood solution of the approximated gaussian rate function, i.e.

- The initial point is $\mathbf{u}_0 = \text{argmax}\ \chi^2_{\text{L}}(\mathbf{u})$

- The proposed point is $\mathbf{u}_{l+1,p} = \mathbf{u}_l + \delta_k \mathbf{C}_k$, where $\mathbf{C}_k$ is the k-th eigenvector and δ_k is a gaussian random variable of zero mean and standard deviation equal to the k-th eigenvalue upon diagonalizing the inverse of the flux covariance matrix in gaussian approximation. The number k is selected uniformly at random.

This reduces sampling times by a factor approximatively equal to the conditioning number, i.e. the ratio between the largest and smallest eigenvalue of the covariance matrix, that in our case is around 10^4, parelling similar analysis for the bulk metabolic networks.[S8] The maximum likelihood estimate has been retrieved finally via simulated annealing,[S9] i.e. multiplying the log-likelihood by a fictitious (inverse) temperature β in the Metropolis scheme, that changes the acceptance probability to $\min(1, e^{\beta \Delta \mathcal{L}})$, and then performing a Montecarlo dynamics with β gradually increasing to high values.

Errors and confidence interval

Once the maximum likelihood estimate for the fluxes has been calculated, their relative errors can be read off from the expression of the log-likelihood itself upon considering its meaning of for the posterior probability through Bayes theorem.[S10] Confidence intervals (δ_-, δ_+) for the fluxes corresponding to $\sim 97.6\%$ probability (2σ for the Gaussian) have been estimated by looking at flux variations that change accordingly the log-likelihood

$$\mathcal{L}_{\max} - \mathcal{L}(\mathbf{u}_{-i}, u_{i,\mathrm{opt}} \pm \delta_{\pm,i}) = -2 \qquad (25)$$

while for the error bars we use $\Delta \mathcal{L} = -0.5$ and symmetrize. This follows from standard theory on non-parabolic error and confidence interval estimates.[S11]

The error on pairwise flux exchange can be propagated from single cell fluxes through the formula and assuming for simplicity independent errors for the fluxes we have the formula

for the relative errors ($\epsilon_i = \delta_i/|u_i|$)

$$\epsilon_{i\to j} = \epsilon_i + \epsilon_j + \sum_{k:u_k<0} \epsilon_k \phi_{ik}, \qquad \text{where} \quad \phi_{ik} = \frac{u_k/d_{ik}}{\sum_{l:u_l<0} u_l/d_{il}} \tag{26}$$

We expect that exchange fluxes of cells located where the density of probes is higher are bound to be estimated more precisely than those of cells lying in an area with a lower density of probes. This effect is especially clear if one compares errors of exchange fluxes for cells close to the center of the visual field vs near the boundary of the frame as we report in figure S5.

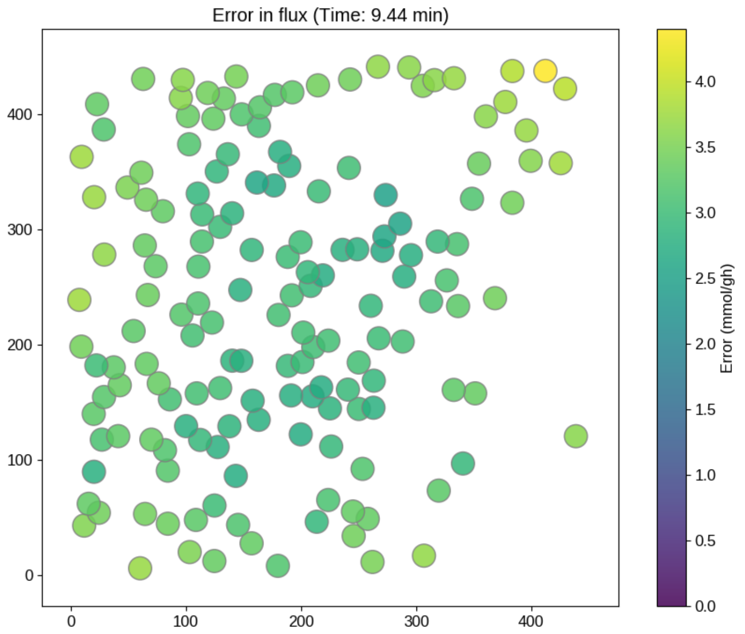

Figure S5: Absolute error on inferred fluxes as a function of cell position in the first analyzed frame.

Ruling out flow contributions from advection

In the most general case, the proton concentration obeys the conservative advection-diffusion equation

$$\frac{\partial c}{\partial t} = \nabla \cdot \vec{f}_{\mathrm{D}} - \nabla \cdot \vec{f}_{\mathrm{A}} \tag{27}$$

where

$$\vec{f}_{\mathrm{D}} = D\nabla c \quad , \quad \vec{f}_{\mathrm{A}} = \boldsymbol{v}c \ \ . \tag{28}$$

Within the manuscript we assumed that the concentration is quasi-stationary (i.e. $\frac{\partial c}{\partial t} = 0$), that the advection currents $\vec{f}_{\mathrm{A}}$ are negligible w.r.t. the diffusion currents $\vec{f}_{\mathrm{D}}$ and also that the medium is homogeneous and isotropic. Under these assumptions, its diffusion coefficient D is constant and this allows us to reduce the advection-diffusion equation to the Laplace equation $\nabla^2 c = 0$ discussed in the manuscript. To prove that advection currents are indeed negligible, we can look at the ratio R of the advection current to the diffusion current (if $R \ll 1$, then the assumption is well justified). By definition,

$$R := \frac{\|f_A\|}{\|f_D\|} = \frac{\|\boldsymbol{v}c\|}{\|D\nabla c\|} = \frac{\|\boldsymbol{v}\|\, c}{D\,\|\nabla c\|} \ . \tag{29}$$

So, in order to estimate R we have to estimate in turn $\|\boldsymbol{v}\|$ (the magnitude of the velocity field that the protons are moving with), the concentration of protons c, the magnitude of its gradient $\|\nabla c\|$ and the diffusion coefficient D). Each of these quantities can be inferred from available data:

- $\|\boldsymbol{v}\|$ can be taken to be approximately equal to the average magnitude of the relative velocities between cells $\|\boldsymbol{v}\| \approx 0.1\,\mu\mathrm{m/min}$. Indeed, keeping in mind that we are using velocities measured in the center-of-mass reference (i.e. for the generic cell i, $v_i = V_i - V_{\mathrm{com}}$, where com stands for center of mass), the relative velocity among two generic cells (i, j) is $v_{ij} = v_i - v_j$. From figure S6b we see that the velocity distribution peaks at around $0.06\,\mu\mathrm{m/min}$ thus $\|v_i - v_j\| < \|v_i\| + \|v_j\| \approx 0.1\,\mu\mathrm{m/min}$).

- D is the diffusion coefficient of protons in water, i.e. $D \sim 7000\,\mu\text{m}^2/\text{s}$

- The estimation of the (mean) proton concentration is more involved. We first observe that most of our pH probes have measured values around pH $= 6.5 \pm 0.5$, this in turn implies that

  - c is on average equal to $10^{-6.5} \approx 3 \times 10^{-7}$, (i.e. the concentration of protons at pH $= 6.5$)
  - the variations of c in magnitude are expected to be around $\delta c \approx |10^{-6} - 10^{-7}|$

  We also observe that within our dataset the average distance δr between the nuclei of neighboring cells is around $35\,\mu\text{m}$ (see figure S6d, specifically the value for $n = 1$). Hence we can estimate the magnitude of the gradient of c as

$$\|\nabla c\| \approx \frac{\delta c}{\delta r} \approx 2.5 \times 10^{-8}\,\mu\text{m}^{-1}. \tag{30}$$

Putting things together we get

$$R = \frac{\|\boldsymbol{v}\|\, c}{D\,\|\nabla c\|} \approx \frac{0.1\,\mu\text{m}/\text{min} \times 3 \times 10^{-7}}{7000\,\mu\text{m}^2/\text{s} \times 2.5 \times 10^{-8}\,\mu\text{m}^{-1}} \tag{31}$$

$$\approx \frac{0.1 \times 3 \times 10^{-7}}{7000 \times 60 \times 2.5 \times 10^{-8}} \approx 3 \times 10^{-6} \ll 1\ . \tag{32}$$

We therefore can safely discard the advective contribution within the advection-diffusion equation, proving that the assumptions within the manuscript are correct. To strengthen the above result, one can reason as follows. If transport were mainly due to advection this would reasonably give rise to a correlation among velocities (in the center of mass framework) such that the correlation length should roughly match the effective radius of the advective transport. Such correlations can however be quantified from data. Figure S6a shows a scatter plot of v_x and v_y that essentially returns a purely diffusive picture (confirmed by a Pearson coefficient roughly equal to $\rho \sim 0.02$); furthermore, in panel S6(b) we show the distribution

of the velocities whose fit is again in agreement with pure diffusion. Panels S6(c,d) display instead the metric correlation function (c) and the topological distance (d). The metric correlation $C(r)$ between the directions $\hat{d}_i$ and $\hat{d}_j$ of two generic cells (i, j) (with $\hat{d}_i = \frac{\boldsymbol{v}_i}{\|\boldsymbol{v}_i\|}$ and $\hat{d}_j = \frac{\boldsymbol{v}_j}{\|\boldsymbol{v}_j\|}$) is defined as

$$C(r) = \frac{\sum_{i,j} \hat{d}_i \cdot \hat{d}_j \delta(r - r_{ij})}{\sum_{i,j} \delta(r - r_{ij})} \ . \tag{33}$$

One sees (panel S6(c)) that $C(r)$ decays rapidly to a single cellular distance, while the number of nearest neighbours grows as $n \sim r^2$ (panel S6(d)) as expected in standard thermal equilibrium in $2D$ (i.e. the topological correlations are in agreement with an equilibrium picture). Finally, panel S6(e) rules out the possibility that cells form lumps or small clusters, while panel S6(f) shows an effective comparison between the advective and diffusive contributions to proton transport by displaying the distribution of the ratio R defined above and estimated as discussed. In summary, also from this perspective the assumption of pure diffusion seems to be suitably justified.

Flux distributions conditioned on the cell type

Our quantification of single cell fermentation fluxes in a co-culture of tumoral and stromal cells could potentially shed new light on the current debate on the role of the cell type in the acidic exchange (stromal vs tumoral). Formally we find that the single cell flux distribution conditioned to the cell type does not lead to detectable differences: the mean flux for the stromal cells is 0.48 mmol/gdw/h and while for the tumoral cells is 0.41 mmol/gdw/h, both with a standard deviation of around 3 mmol/gdw/h, and the histograms superimpose (see figure S7). We thus do not detect any difference between stromal and tumoral cells with regard to the flux values, i.e. their intensity and sign, at least with our resolution and we cannot really provide any support for theories claiming one cell type is preferentially having one role in the putative lactate exchanges.

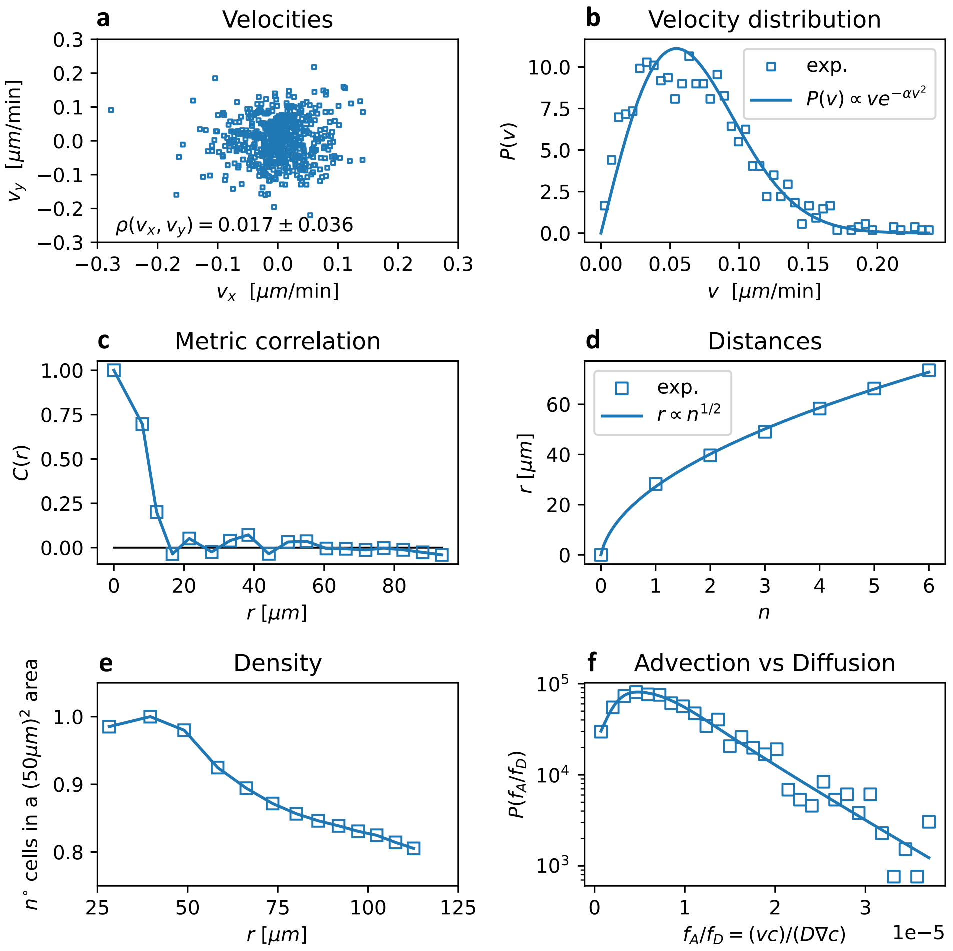

Figure S6: Kinematic properties of cells in the center of mass frame. (a) Cellular velocities (the Pearson correlation coefficient ρ is consistent with 0). (b) Magnitude of the cellular velocities distribution (line: gaussian fit). (c) Metric correlation function (correlation length $\xi \sim 20\,\mu m$. (d) The average distance $r(n)$ of each cell to its n-th neighbor vs n, (line: fit of the scaling $r \propto n^{1/2}$, confirming homogeneity) (e) Density of cells vs length-scale r) (f) Distribution of the ratio among the advective current f_A and the diffusive current f_D.

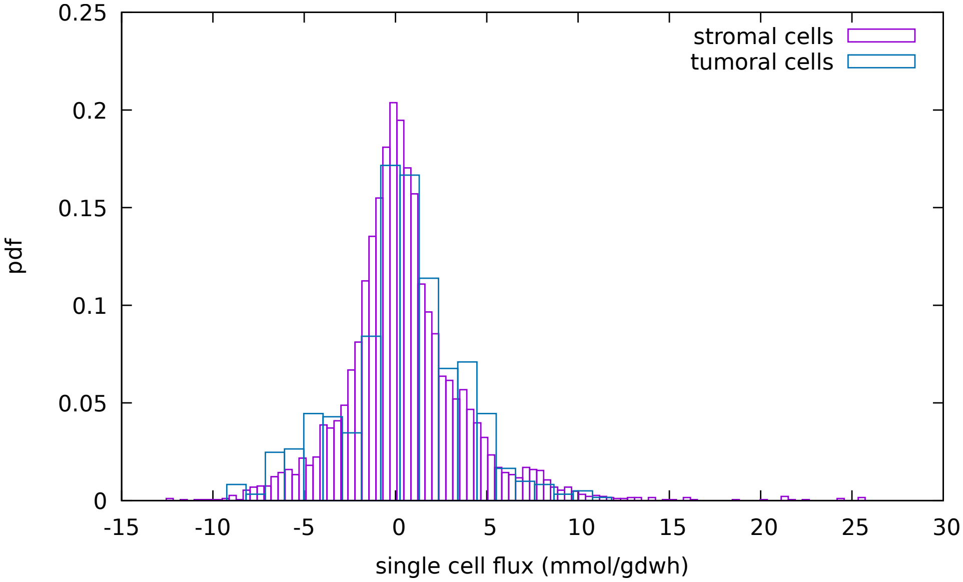

Figure S7: Single cell flux distributions conditioned to the cell type (tumoral vs stromal) across all frames.

pH maps

We showcase in figure S8, the reconstructed pH maps, specifically for the frame number 1, 10 and 20. The pattern is interesting and relatively complex: in a bulk of acidic condition cells (mainly stromal) seem able to maintain and extend a neutral patch. It seems the latter is surrounded by a boundary with a "depletion zone" (highlighted here in blue, meaning very low density of protons probably due to high influx from the cells inside the patch). We report however that our error on such basic pH values is rather high (it is almost in the outside the linear region of the calibration curve of the probes).

Supporting files description

The following files can be found at `https://github.com/demartid/infer_single_cell_fermentation_codes_data`

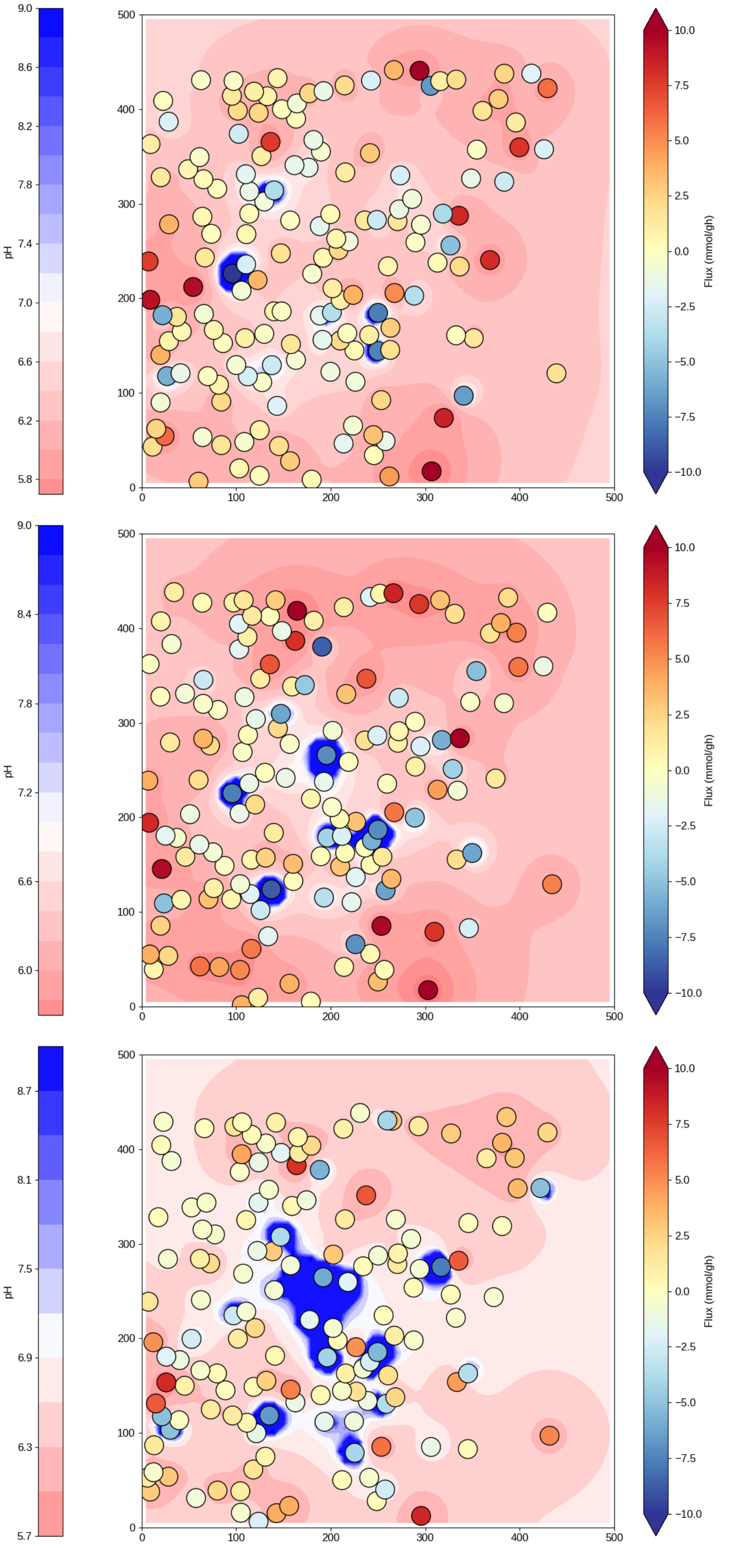

Figure S8: Reconstructed pH maps for the frames 1, 10, 20.

Data

We provide two data files:

- `probes.dat` reports the position and measured pH value, with error for each probe at given timepoints.
- `cells.dat` reports the position and measured flux, with error and confidence intervals, for each cell at given timepoints.

Codes

We provide two codes:

- `gauss.py` takes as input the positions and pH values (with errors) of probes and the positions of cells for a given frame and gives as output the maximum likelihood estimate of the cell fluxes and their covariance matrix in gaussian approximation.
- `sim_ann.cpp` accepts as input the inverse distance matrices of cells and probes for all frames, the gaussian estimated flux and covariance, the cell tracking and it gives as output the maximum likelihood estimate of the cell fluxes with errors and the confidence interval.

Supplementary Figures

We report here in the figures S9–S14 the map of the inferred single cell fluxes and intercellular exchanges (Arrows are added between cells whose exchange exceeds our average sensitivity 0.5 mmol/gdw/h) for all the 36 time frames, and in the figures S14–S18 the associated quality of the reconstructed pH gradient profile. In the latter the error between the pH calculated from the inferred fluxes and the experimentally observed pH is plotted against the latter for each probe for all the observed time frames.

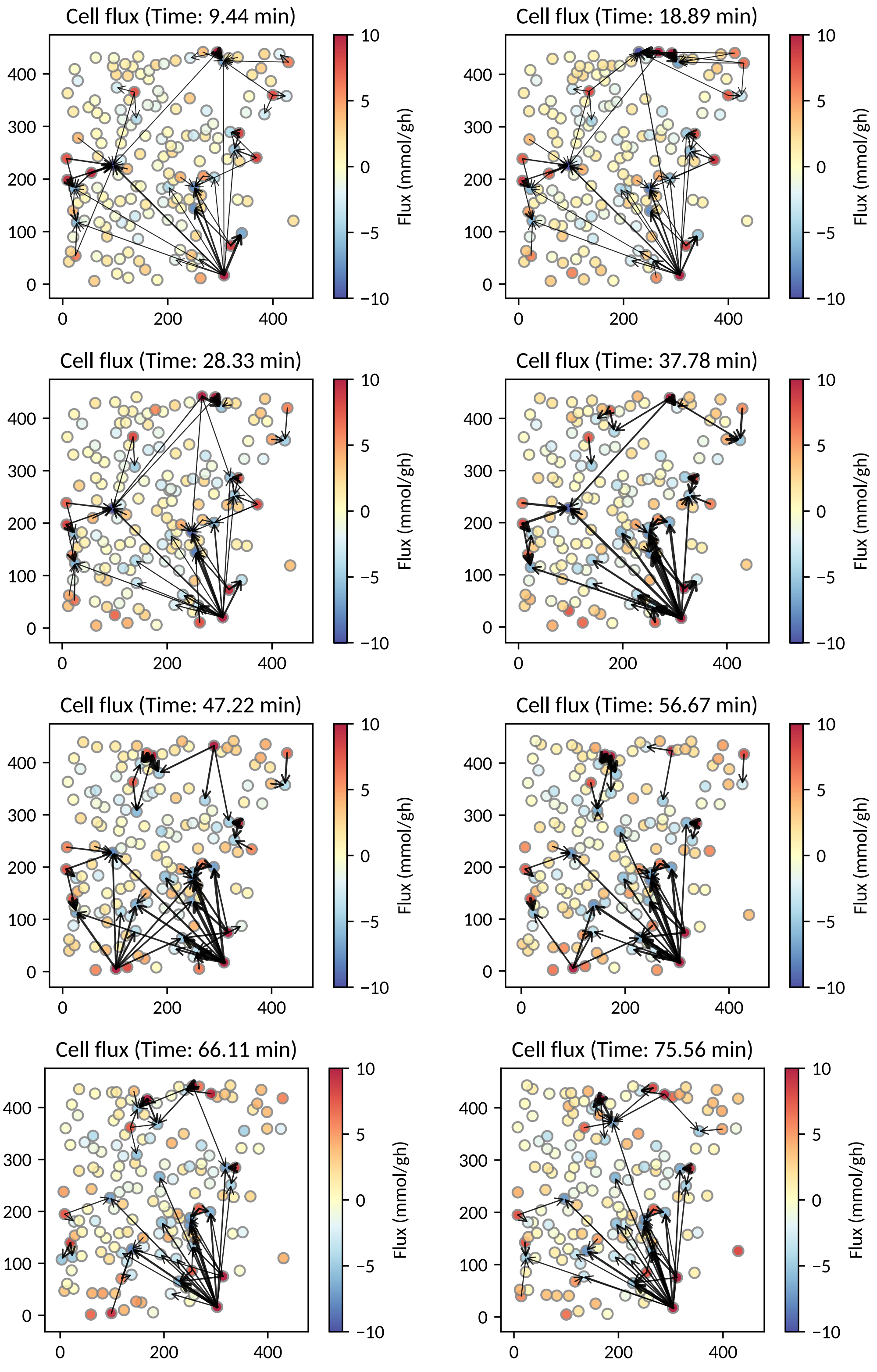

Figure S9: Cell fluxes for time $t < 1\,\mathrm{h}\ 20\,\mathrm{m}$

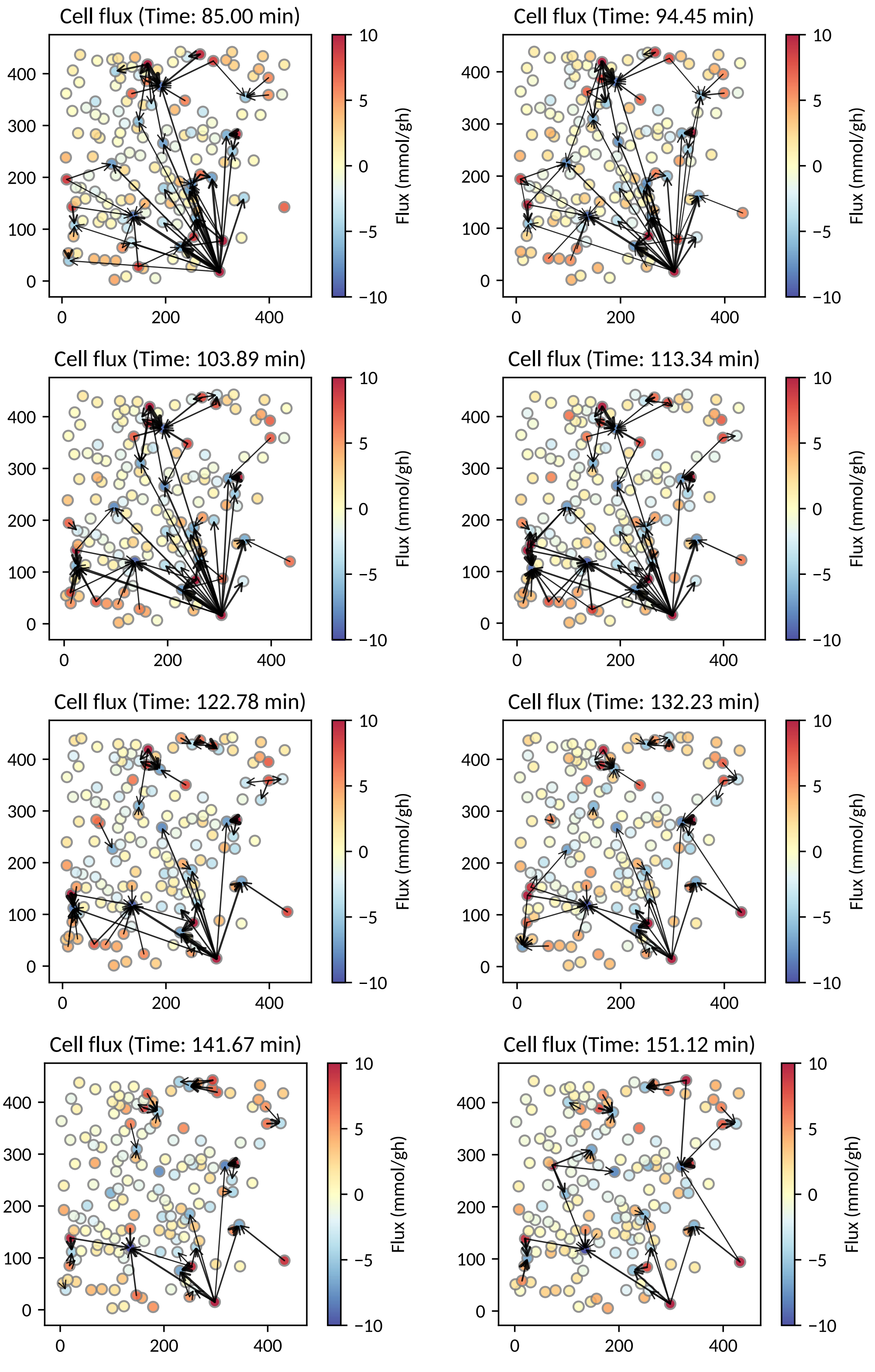

Figure S10: Cell fluxes for time 1 h 20 m $< t <$ 2 h 30 m

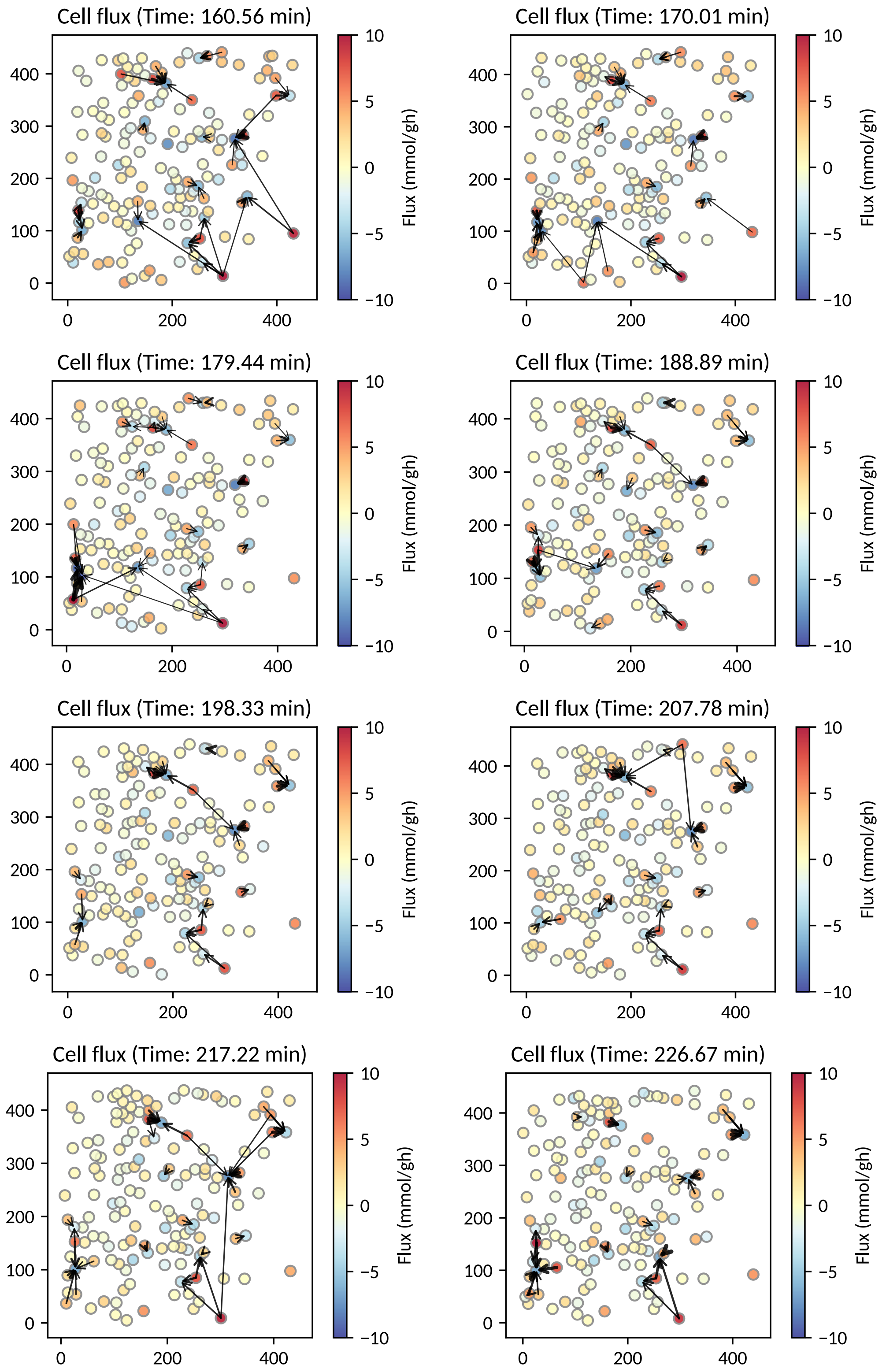

Figure S11: Cell fluxes for time 2 h 30 m < t < 3 h 45 m

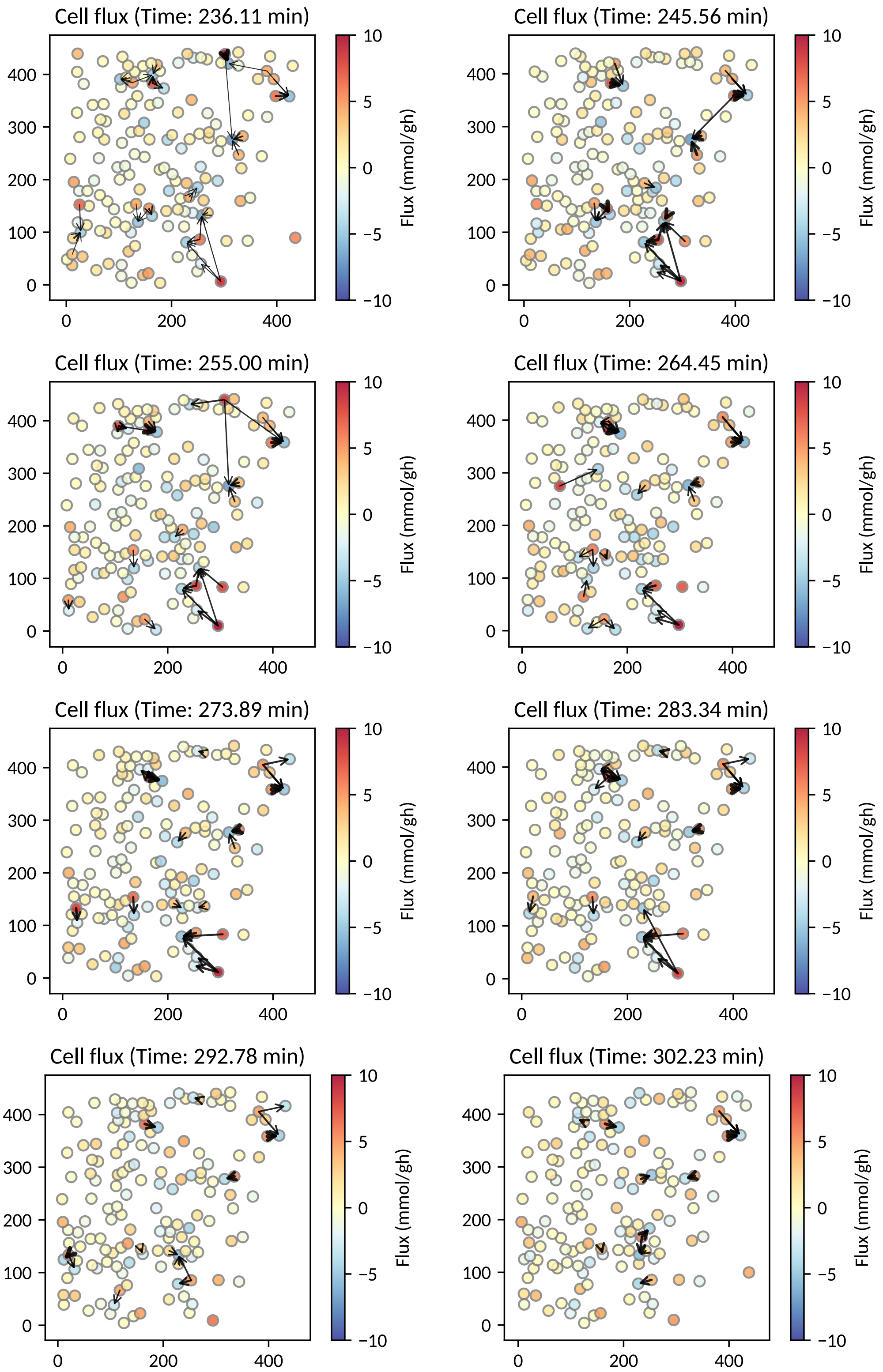

Figure S12: Cell fluxes for time $3\,\mathrm{h}\ 45\,\mathrm{m} < t < 5\,\mathrm{h}$

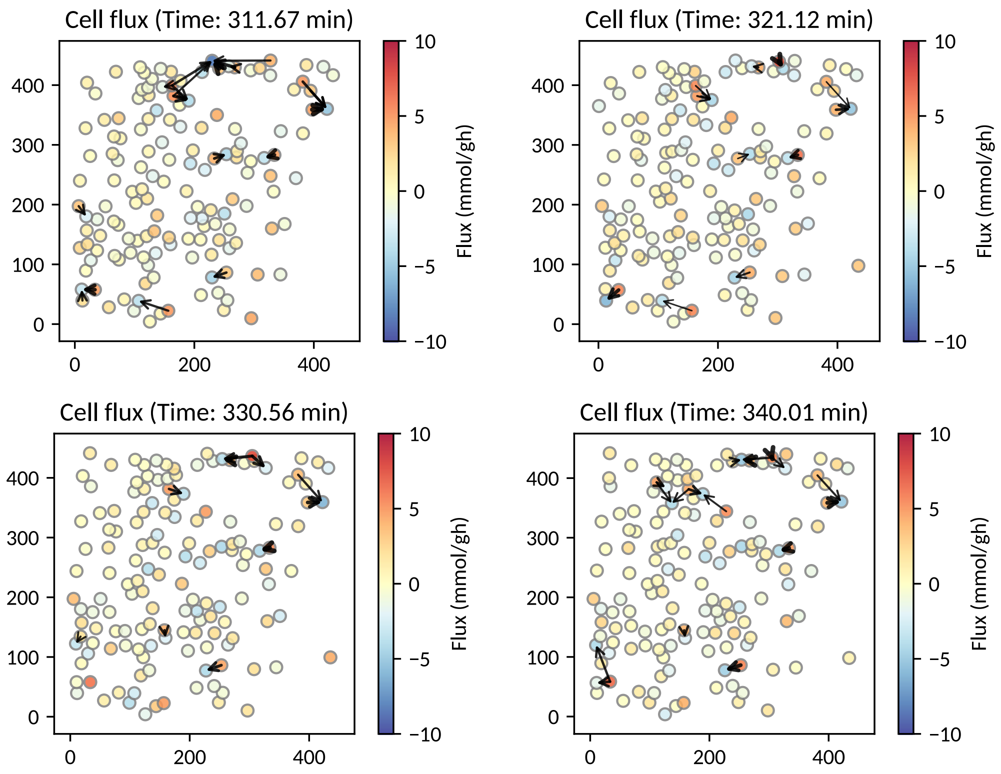

Figure S13: Cell fluxes for time $t > 5\,\mathrm{h}$

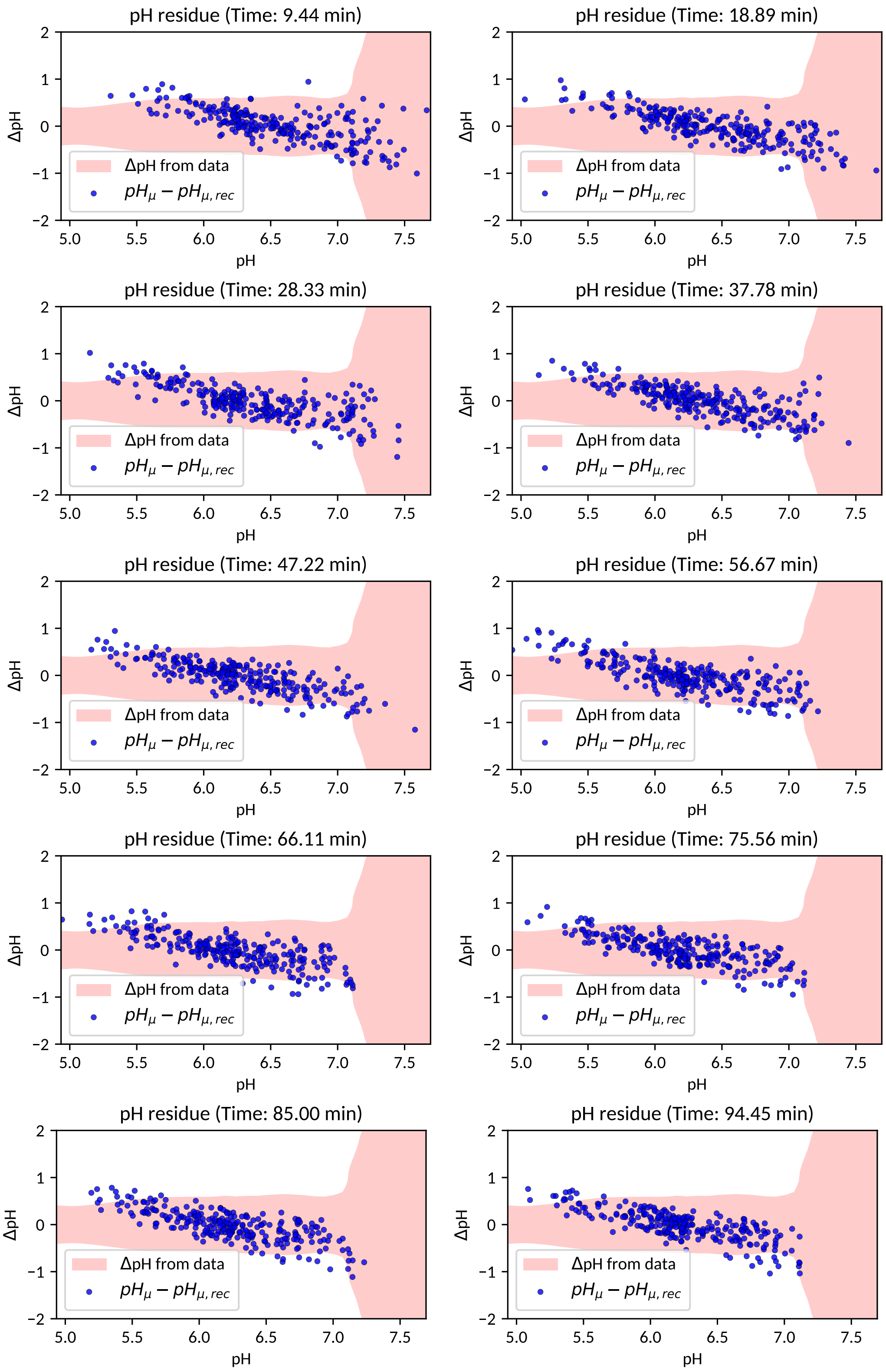

Figure S14: Cell fluxes for time $t < 1\,\mathrm{h}\ 35\,\mathrm{m}$

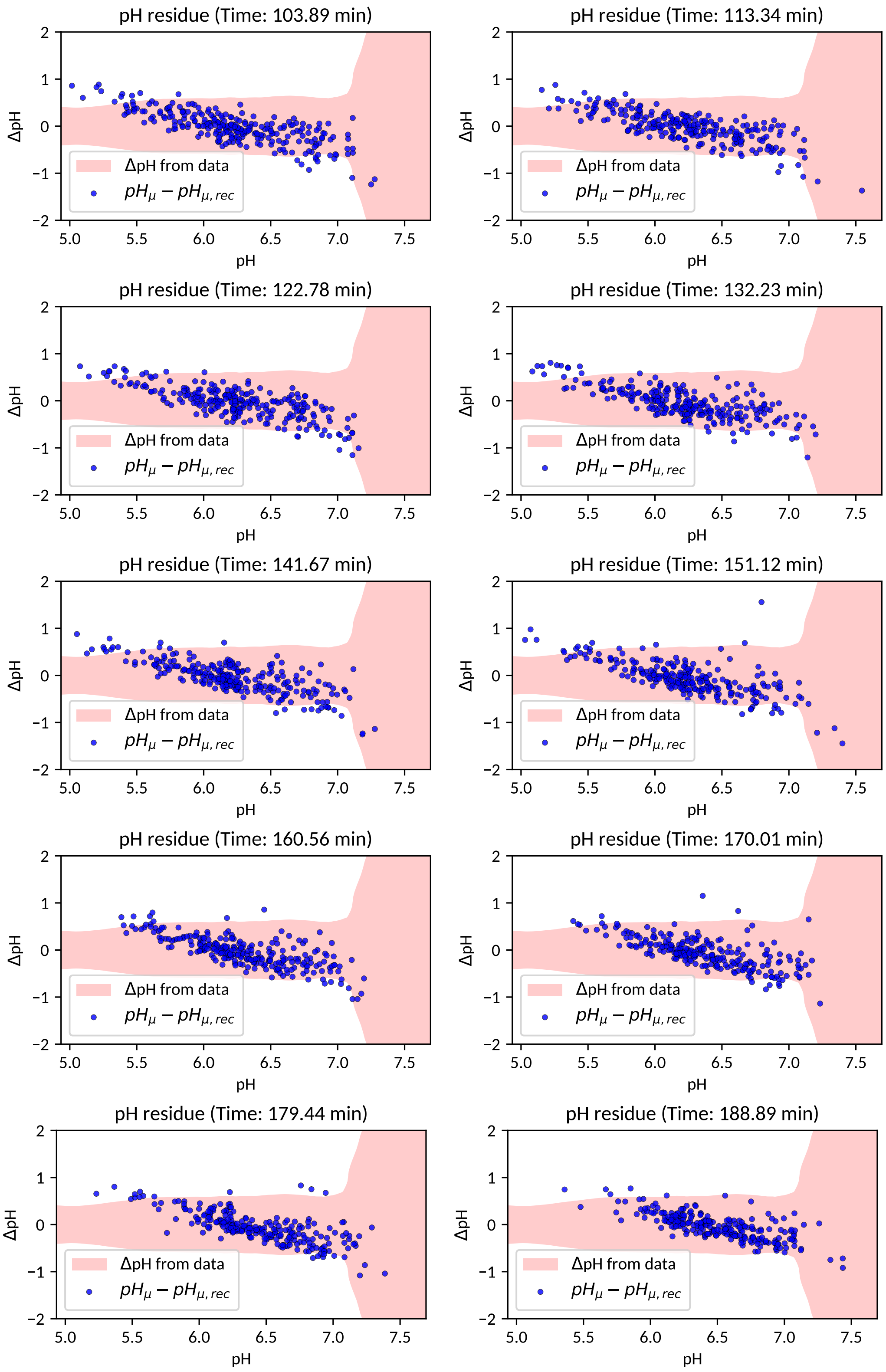

Figure S15: Cell fluxes for time $1\,\mathrm{h}\ 45\,\mathrm{m} < t < 3\,\mathrm{h}\ 10\,\mathrm{m}$

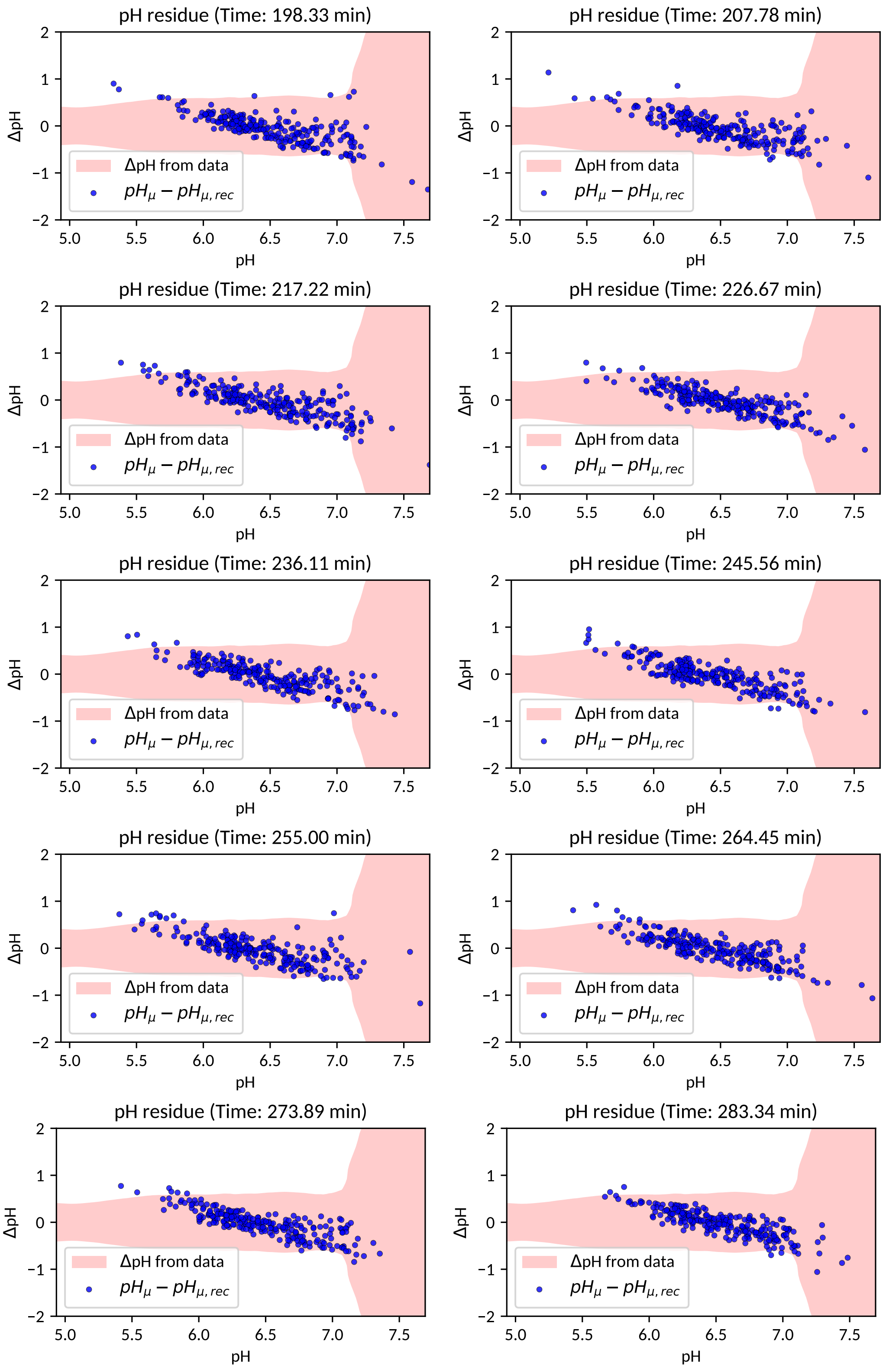

Figure S16: Cell fluxes for time $3\,\mathrm{h}\ 20\,\mathrm{m} < t < 4\,\mathrm{h}\ 45\,\mathrm{m}$

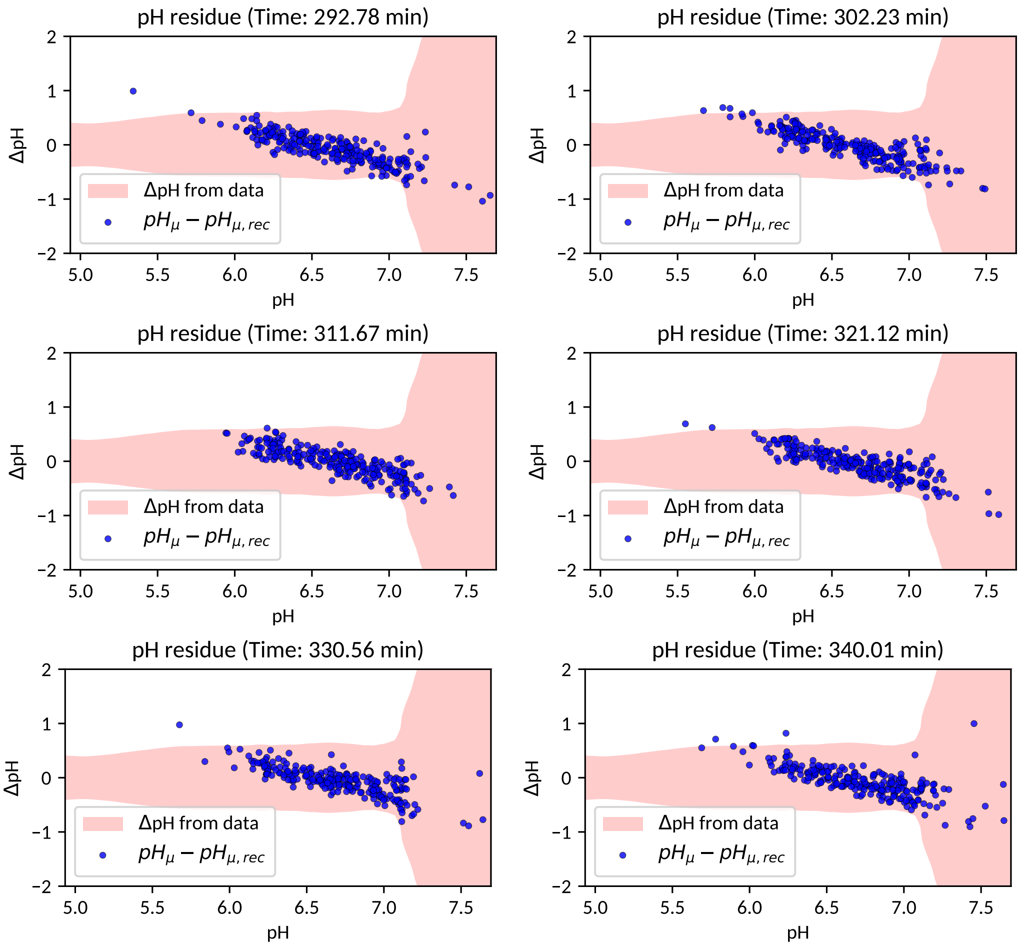

Figure S17: Cell fluxes for time $t > 4\,\mathrm{h}\ 45\,\mathrm{min}$

References

(S1) Chandra, A.; Prasad, S.; Alemanno, F.; De Luca, M.; Rizzo, R.; Romano, R.; Gigli, G.; Bucci, C.; Barra, A.; del Mercato, L. L. Fully automated computational approach for precisely measuring organelle acidification with optical ph sensors. *ACS Applied Materials & Interfaces* **2022**, *14*, 18133–18149.

(S2) Schneider, C. A.; Rasband, W. S.; Eliceiri, K. W. NIH Image to ImageJ: 25 years of image analysis. *Nature Methods* **2012**, *9*, 671–675.

(S3) Wu, H.; Ying, M.; Hu, X. Lactic acidosis switches cancer cells from aerobic glycolysis back to dominant oxidative phosphorylation. *Oncotarget* **2016**, *7*, 40621–40629.

(S4) Shih, F. Y. *Image Processing and Mathematical Morphology: Fundamentals and Applications*; CRC press, 2017.

(S5) Jaqaman, K.; Loerke, D.; Mettlen, M.; Kuwata, H.; Grinstein, S.; Schmid, S. L.; Danuser, G. Robust single-particle tracking in live-cell time-lapse sequences. *Nature Methods* **2008**, *5*, 695–702.

(S6) Goldfarb, D.; Idnani, A. A numerically stable dual method for solving strictly convex quadratic programs. *Mathematical Programming* **1983**, *27*, 1–33.

(S7) Jackson, V. N.; Halestrap, A. P. The Kinetics, Substrate, and Inhibitor Specificity of the Monocarboxylate (Lactate) Transporter of Rat Liver Cells Determined Using the Fluorescent Intracellular pH Indicator. *Journal of Biological Chemistry* **1996**, *271*, 861–868.

(S8) De Martino, D.; Mori, M.; Parisi, V. Uniform sampling of steady states in metabolic networks: heterogeneous scales and rounding. *PloS One* **2015**, *10*, e0122670.

(S9) Kirkpatrick, S.; Gelatt Jr, C. D.; Vecchi, M. P. Optimization by simulated annealing. *Science* **1983**, *220*, 671–680.

(S10) MacKay, D. J. *Information Theory, Inference, and Learning Algorithms*; Cambridge university press, 2003.

(S11) James, F. *Statistical Methods in Experimental Physics*; World Scientific Publishing Company, 2006.
